# Vinyl-pyrazole as a biomimetic acetaldehyde surrogate†

**DOI:** 10.1039/d4cc01305k

**Published:** 2024-06-04

**Authors:** Lorenz Steiner, Miljan Z. Ćorović, Antoine Dupé, Nadia C. Mösch-Zanetti

**Affiliations:** a Institute of Chemistry, Inorganic Chemistry, University of Graz 8010 Graz Austria nadia.moesch@uni-graz.at

## Abstract

Inspired by the enzyme acetylene hydratase, we investigated the reactivity of acetylene with tungsten(ii) pyrazole complexes. Our research revealed that the complex [WBr_2_(pz-NHCCH_3_)(CO)_3_] (pz = 3,5-dimethyl-pyrazolate) facilitates the stochiometric reaction between pzH and acetylene to give *N*-vinyl-pz. This vinyl compound readily hydrolyzes to acetaldehyde, mirroring the product of acetylene hydration in the enzymatic process. The formation of the vinyl compound likely involves a reactive intermediate complex where acetylene acts as a two-electron donor, in contrast to isolable acetylene complexes that are inert to nucleophilic attack by water. Results suggest an alternative mechanism for the enzyme, including vinylation of a neighboring amino acid by acetylene in the active site prior to hydration.

## Abstract 2

Acetaldehyde formation is mediated by a tungsten pyrazole complex *via* a vinyl intermediate and subsequent hydrolysis—a rare example of the reactivity of unsubstituted acetylene.

## Graphical abstract

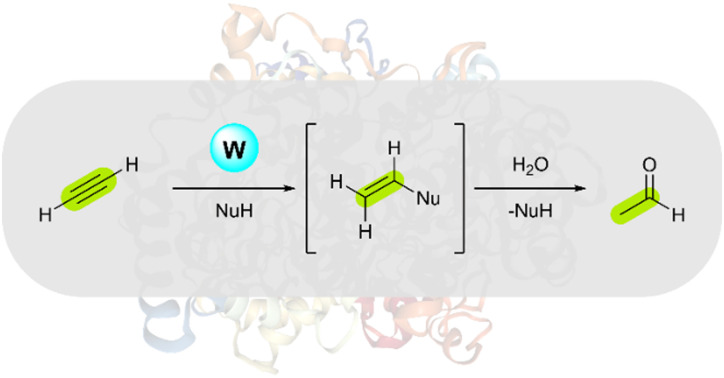

Acetylene hydratase (AH) found in *Pelobacter acetylenicus* is a tungstoenzyme catalyzing the hydration of acetylene.^1^ X-ray diffraction analysis of the enzyme revealed the active site consisting of a tungsten(iv) center coordinated to two sulfur-rich metallopterine moieties, a cysteine residue (Cys141), and a water/hydroxide ligand (Scheme 1). Adjacent to the active site, a critical aspartate residue (Asp13) was identified in the second coordination sphere.^2^

**Scheme 1:**
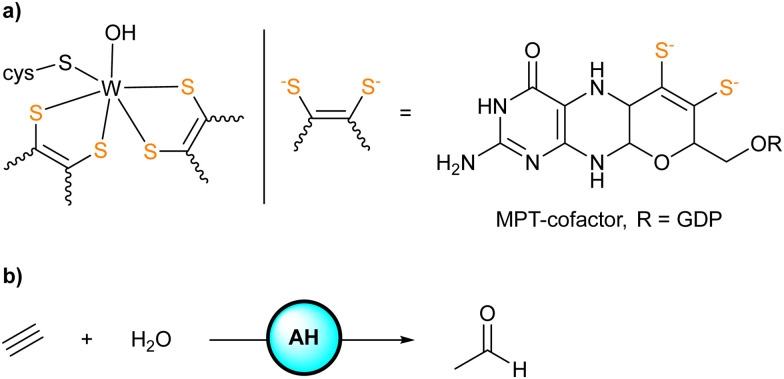
(a) Representation of the active site of AH (GDP = guanosine diphosphate). (b) Hydration of acetylene catalyzed by AH to give acetaldehyde.

The role of the tungsten center is the subject of an ongoing debate about the mechanism.^3^ Theoretical studies have explored two main classes of mechanistic approaches: one proposing acetylene coordination followed by nucleophilic attack of water, and the other suggesting coordinated water/hydroxide attacking acetylene in the second coordination sphere.^4–10^ Notably, both mechanisms involve the coordination of a substrate (acetylene or water) and direct reaction with the other non-bound substrate. Biomimetic modeling of acetylene hydratase (AH) is scarce. Dithiolene ligands have been used for the modeling of the MPT cofactor^11^ in tungstoenzymes which led to the development of oxygen and sulfur atom transfer catalysts,^12–14^ and recently to the reduction of CO_2_ to formate.^15^ No tungsten acetylene complexes are known to facilitate acetylene hydration since a functional model has been recently disproven.^16,17^ Only a high-valent polynuclear tungsten species is known to catalyze higher alkyne hydration, albeit under extreme conditions.^18^

Our group developed tungsten(ii) and tungsten(iv) pyridine–thiolate complexes of the type [W(CO)(C_2_H_2_)(R-PyS)_2_] to mimic the W–C_2_H_2_ intermediate suggested by theoretical studies.^19,20^ While lower oxidation state complexes readily undergo acetylene insertion into the W–N bond of the ancillary ligand,^21^ such acetylene activation has not been observed for tungsten(iv) species. In contrast, a reaction involving the [MoO(C_2_H_2_)(6-MePyS)_2_] complex (6-MePyS = 6-methylpyridine-2-thiolate) with acetylene and water resulted in the formation of 6-methyl-1-vinyl-pyridine-2(1*H*)-thione.^22^ Although it is conceivable that such a species could undergo hydrolysis to yield acetaldehyde and the free ligand, this behavior has not been observed. To obtain acetaldehyde from a tungsten acetylene complex and water, we screened our tungsten complexes containing pyridine-2-thiolates as well as the tungsten(ii) scorpionate complex [Tp*WI(CO)_3_] (Tp* = hydridotris(3,5-dimethylpyrazolyl)borate) reported by Templeton and coworkers in the presence of acetylene and water.^23^ Only with the latter we observed traces of acetaldehyde alongside the decomposition of the starting complex to *N*-vinyl-3,5-dimethyl-pyrazole and other unidentified compounds. The occurrence of a vinyl-pyrazole is noteworthy as direct vinylation of pyrazole with acetylene is rare and proceeds under harsh conditions.^24^

Therefore, we aimed to simplify the initial scorpionate ligand to 3,5-dimethyl pyrazole (pzH) and probed a tungsten complex thereof for acetylene hydration.

As described in the ESI,† compound [WBr_2_(CO)_3_(pzH)_2_] (1) (pzH = 3,5-dimethylpyrazole) was obtained from [WBr_2_(CO)_3_(MeCN)_2_] and 2 equiv. of pzH in CH_2_Cl_2_ solution as orange crystals in 84% yield (Scheme 2). ^1^H NMR spectroscopy reveals a single isomer in solution, with only one ligand set due to the “propeller” effect of the tricarbonyl moiety. IR stretching frequencies of the tricarbonyl moiety are in the range of 2023–1885 cm^−1^ and follow other tungsten(ii) tricarbonyl complexes.^19^ XRD analysis suitable single crystals were obtained from CH_2_Cl_2_/*n*-heptane solution at −30 °C. The analysis confirms a hepta-coordinated tungsten(ii) center with three carbonyl groups in facial arrangement, two bromides in *cis* position, and two protonated monodentate pyrazole ligands which is similar to previously reported [WI_2_(CO)_3_(pzH)_2_].^25^ Dissolving 1 in acetonitrile and stirring the solution for 4 h at rt leads to decoordination of one pzH ligand and quantitative formation of [WBr_2_(pz-NHCCH_3_)(CO)_3_] (1i) *via* insertion of the solvent into the W–N bond of the other deprotonated pyrazolate ligand (Scheme 2 and Fig. S13, ESI†). Compound 1i is soluble in acetonitrile and dichloromethane. Compared to 1, the IR stretching frequencies of the tricarbonyl moiety are higher in 1i (2033–1899 cm^−1^) as a consequence of less pronounced π-backdonation.

**Scheme 2:**
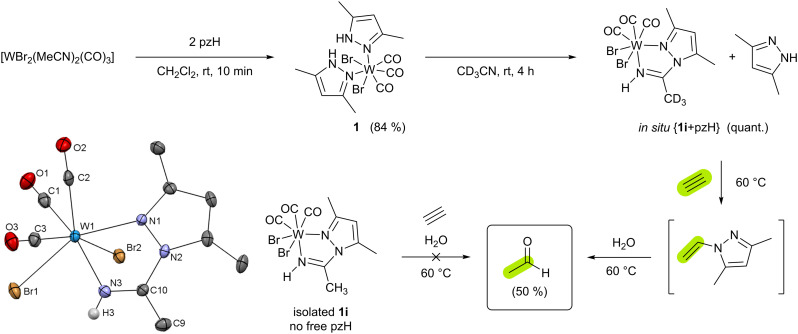
Synthesis of complexes 1 and 1i, respectively. Formation of acetaldehyde from acetylene and water is mediated by 1i*via* intermediate *N*-vinyl-3,5-dimethyl-pyrazole only when prepared *in situ*. Isolated pzH-free solutions of 1i do not give acetaldehyde under the same conditions. Molecular structure of 1i. The ellipsoids are drawn at the 50% probability level. Solvent molecule is omitted for clarity. Except for the one at nitrogen the H atoms were omitted for clarity.

To test the acetylene hydration activity, a solution of 1 in CD_3_CN and mesitylene (internal standard) was allowed to fully convert to 1i and pzH before mixing it with excess acetylene and H_2_O (5 equiv.). Heating this mixture at 60 °C for 4 h gives acetaldehyde in 50% yield (Scheme 2 and Fig. S14, ESI†) as the major product. If compound 1 is dissolved in MeCN and mesitylene, water, and acetylene are added immediately, the amount of acetaldehyde obtained is much lower (5%) (Fig. S15, ESI†). Most likely, the decomposition of 1 in water competes with the formation of 1i. No reaction occurs between acetylene and pzH and/or water in absence of tungsten. Labeling experiments with deutero-acetylene C_2_D_2_ lead to the formation of 1,2-dideutero-acetaldehyde as demonstrated by the absence of lowfield resonances in the aldehyde region of the ^1^H NMR spectrum (Fig. S16, ESI†), as well as the presence of a characteristic triplet expected for deuterium labeled –CH_2_D moiety. The presence of deuterated aldehyde was additionally confirmed by HPLC-MS (see ESI†). Monitoring the reaction in CD_3_CN in time intervals by ^1^H NMR spectroscopy revealed the intermediate formation of *N*-vinyl-3,5-dimethyl-pyrazole^26^ (Fig. S17, ESI†) which is subsequently converted to acetaldehyde and pzH. Indeed, in absence of water, only *N*-vinyl-3,5-dimethyl-pyrazole is observed which converts to acetaldehyde after the addition of water (Fig. S18, ESI†). However, we found that *N*-vinyl-3,5-dimethyl pyrazole may also be hydrolyzed to acetaldehyde in the absence of tungsten but upon the addition of mineral acids (Fig. S19, ESI†).

From the *in situ* solution of {1i + pzH}, the former can be separated by crystallization. The reaction of pure 1i in MeCN with water and acetylene does not yield acetaldehyde, as confirmed with ^1^H NMR experiments emphasizing the role of one additional pzH. However, the addition of an excess of pzH to the *in situ* mixture {1i + pzH} in CD_3_CN followed by the addition of acetylene and water, decreases the acetaldehyde yield (16%). Furthermore, the ^1^H NMR spectrum revealed the formation of the cationic acetylene complex [WBr(C_2_H_2_)(pzH)(pz-NHCCH_3_)(CO)]Br (2i, 25%; Fig. S20, ESI†) where an additional pzH is coordinated. The latter was independently synthesized *via* an alternative route (*vide infra*) and was found to be unreactive towards acetylene hydration.

Generally, small amounts of benzene and crotonaldehyde were identified as byproducts by ^1^H NMR spectra in all of the reactions containing tungsten complexes 1 or 1i, water, and acetylene (Fig. S14, ESI†). Most likely, benzene is formed as the cyclotrimerization product of acetylene, while the latter could either originate from acetaldehyde (*via* aldol condensation) or acetylene (*via* oxidative coupling of two acetylene molecules, followed by protonation and subsequent hydration). The aldol reaction pathway is ruled out as heating a CD_3_CN solution of {1i + pzH}, H_2_O (5 equiv.), and acetaldehyde (2 equiv.) at 60 °C for 4 h did not result in the formation of crotonaldehyde, as confirmed by ^1^H NMR spectroscopy (Fig. S21, ESI†).

To get insight into the mechanism of acetylene hydration, the reactions of 1 or {1i + pzH} with acetylene were performed under dry conditions, however leading to intractable mixtures, independent of the solvent used. The only possibility to isolate an acetylene complex with the tungsten(ii) pyrazole moiety was adding the pzH ligand to a tungsten precursor already containing coordinated acetylene. Therefore, combining the acetylene precursor mixture [WBr_2_(C_2_H_2_)(MeCN)_2_(CO)]/[WBr_2_(C_2_H_2_)_2_(MeCN)(CO)]^21^ with 2 equiv. of pzH *vs.* W in CH_2_Cl_2_ and subsequent work-up (see ESI†) gave [WBr_2_(C_2_H_2_)(pzH)_2_(CO)] (2), as a bright blue powder in 38% yield (Scheme 3). Similar to 1, 2 quantitatively reacts with acetonitrile to the cationic complex [WBr(C_2_H_2_)(pzH)(pz-NHCCH_3_)(CO)]Br (2i) (Scheme 3). However, flushing solutions of 2 with excess acetylene, leads to a black precipitate, most likely polyacetylene.

**Scheme 3:**
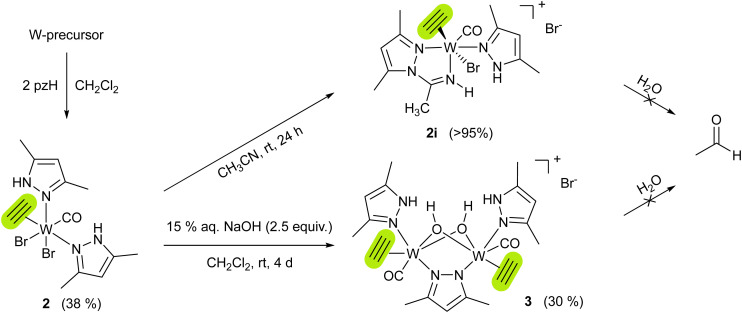
Synthesis of acetylene complexes [WBr_2_(C_2_H_2_)(pzH)_2_(CO)] (2), [WBr(C_2_H_2_)(pzH)(pz-NHCCH_3_)(CO)]Br (2i), and [W(C_2_H_2_)(CO)(pzH)(μ-OH)_2_(μ-pz)W(C_2_H_2_)(CO)(pzH)]Br (3). W-precursor = 1 : 1 mixture of [WBr_2_(C_2_H_2_)(MeCN)_2_(CO)]/[WBr_2_(C_2_H_2_)_2_(MeCN)(CO)]. Complexes 2i and 3 do not react with water to give acetaldehyde.

Both compounds 2 and 2i are well soluble in chlorinated solvents, and THF, but only sparingly soluble in benzene, and insoluble in saturated hydrocarbons. Solutions in chlorinated solvents partly decompose within several days at room temperature. The structure of 2i was confirmed by XRD analysis of crystals obtained from MeCN/Et_2_O at −30 °C which shows an almost octahedral cationic tungsten(ii) complex with a bromide counter anion. Insertion of acetonitrile in the pyrazole N–H bond resulted in the formation of a pyrazolyl-amidino bidentate ligand. The η^2^-acetylene ligand is located *trans* to the bromido ligand with bond lengths similar to other tungsten acetylene complexes (C1–C2 1.301(5) Å, W1–C_2_ 1.914(1) Å),^19^ and acts as a four-electron donor.^27^ The tendency of acetonitrile for insertion into metal-nitrogen bonds has previously been observed in the Re pyrazole complex [ReBr(CO)_3_(pzH)_2_].^28^ According to ^1^H NMR spectra (Fig. S4 and S7, ESI†), acetylenic protons of complexes 2 and 2i resonate in the range of 12.98–13.33 ppm, in line with other tungsten(ii) acetylene complexes from literature.^20,21,29^ The acetylenic protons were distinguished from the pyrazole N–H by comparison with deutero-acetylene analogs of complex 2 and 2i, which were independently synthesized (see ESI†).

Surprisingly, both acetylene complexes 2 and 2i, neutral and cationic, are stable towards water, ruling out the possibility of acetaldehyde formation by nucleophilic attack of water to the W-coordinated acetylene. However, overlaying a CH_2_Cl_2_ solution of 2 with an aqueous solution of NaOH led to the isolation of the μ-hydroxido bridged tungsten(ii) dimer [W(C_2_H_2_)(CO)(pzH)(μ-OH)_2_(μ-pz)W(C_2_H_2_)(CO)(pzH)]Br (3) (Scheme 3) as violet needle-shaped crystals. Its molecular structure was confirmed by single-crystal XRD analysis (Fig. S25, ESI†). Complex 3 is insoluble in most organic solvents as well as in water. It is soluble in DMF and DMSO, solutions of the latter remained stable over several days even at elevated temperatures (60 °C). ^1^H NMR spectrum of 3 in DMSO-*d*_6_ reveals one set of the ligands, confirming the C_2_ rotation axis. Although 3 does not react with water or acetylene, it represents a rare example of a tungsten complex bearing both substrates of the enzyme AH. The only other example represents [Tp*WO(H_2_O)(C_2_H_2_)][OTf], reported by Templeton and co-workers.^30^ None of the models yielded acetaldehyde.

The here reported data show that acetylene hydration mediated by 1i occurs *via N*-vinyl-3,5-dimethyl-pyrazole. *In situ* formed tungsten(ii) compound 1i facilitates the reaction between acetylene and free pzH. Possibly, the reaction occurs over a tungsten intermediate containing both substrates, followed by acetylene insertion into the W–N(pz) bond.

The acetylene ligand in the reactive intermediate must act as a two-electron donor, as such binding is known to lead to vinyl complexes upon nucleophilic attack.^31^ Here, such interaction is enabled *via* substitution of only one carbonyl ligand in 1i. While a tungsten vinyl-pyrazolate complex was not observed, the occurrence of both 1i and 2i strongly supports its formation. In a competitive reaction, two carbonyls can be substituted forming 2i where acetylene ligand acts as a four-electron donor, preventing further reactivity. In general, four-electron donor alkyne complexes only react with extremely potent nucleophiles such as hydrides, organo-alkali reagents, and phosphines.^32^

Released *N*-vinyl-3,5-dimethyl-pyrazole acts as an acetaldehyde surrogate. The subsequent hydrolysis to acetaldehyde and pzH could occur either catalyzed by a W species or by protons released by the decomposition of the W complex, as confirmed experimentally with mineral acid and in the absence of W. Nevertheless, the pyrazole complex 1i represents the first early transition metal compound known to facilitate acetylene hydration, making it a functional model of acetylene hydratase (AH). However, it is not a structural model with respect to the oxidation state and the first coordination sphere. Furthermore, a reactive intermediate with a two-electron acetylene ligand was neither detected in our system nor in AH. Possibly, a similar intermediate is formed in both cases since a stronger, and thus detectable binding would prevent reactivity. A new perspective on the AH mechanism arises including vinylation of a neighboring amino acid in the active site. Acetylene insertion could occur into the W–S or W–O bond of neighboring Cys141 or Asp13 residues before hydrolysis to acetaldehyde. The involvement of amino acid residues that covalently bind to acetylene has not been previously suggested and requires future experimental and computational focus.

Financial support by NAWI Graz and the Austrian Science Fund (grant number P31583) is gratefully acknowledged.

## Conflicts of interest

There are no conflicts to declare.

## Supplementary Material

CC-060-D4CC01305K-s001

CC-060-D4CC01305K-s002
