# Supplementary material for: Vinyl-pyrazole as a biomimetic acetaldehyde surrogate
Source: Chem Commun (Camb). 2024 Jun 4;60(54):6873–6. doi: 10.1039/d4cc01305k (PMC11218809; doi:10.1039/d4cc01305k)
Supplement: CC-060-D4CC01305K-s001 [file CC-060-D4CC01305K-s001.pdf]

Electronic Supplementary Material (ESI) for ChemComm.
This journal is © The Royal Society of Chemistry 2024

Electronic Supplementary Information

Vinyl-pyrazole as a biomimetic acetaldehyde surrogate

Lorenz Steiner,[a] Miljan Z. Ćorović,[a] Antoine Dupé,[a] Nadia C. Mösch-Zanetti,[a]*

[a]Institute of Chemistry, Inorganic Chemistry, University of Graz, 8010 Graz, Austria

* Corresponding author

E-mail address: nadia.moesch@uni-graz.at

Table of Contents

1 General considerations 2

2 Syntheses 4

3 NMR spectra of compounds 7

4 Reactivity studies 19

5 Crystal structure determination 31

6 References 39

1 General considerations

All experiments were carried out under inert atmosphere employing standard Schlenk and glovebox techniques unless otherwise stated. Commercially available chemicals were used as received. Air and moisture-sensitive chemicals were stored in Schlenk flasks or under N_2 atmosphere in a glovebox; liquids were additionally stored over molecular sieves. The tungsten complexes $[WBr_2(MeCN)_2(CO)_3]$[1] and $[WBr_2(C_2H_2)(MeCN)_2(CO)]/[WBr_2(C_2H_2)_2(MeCN)(CO)]$[1,2] were prepared by published procedures using Schlenk and glovebox techniques. C_2D_2 was generated *in situ* by adding D_2O to CaC_2 and was passed through $CaCl_2$ for drying. Oxygen-free solutions of NaOH were obtained by flushing the solution with N_2 for 1 h. All solvents were purified by a Pure Solv Solvent Purification System and stored over activated molecular sieves (3 or 4 Å). NMR spectra were recorded using Bruker Avance III and Bruker Avance NEO 500 MHz spectrometers. ^1H NMR spectra were recorded at 300 MHz for room temperature or at 500 MHz for low-temperature measurements and referenced to residual protons of the NMR solvents. ^{13}C NMR spectra were obtained at 75 MHz and spectra were referenced to the deuterated solvent peak. The chemical shifts δ are given in ppm. The multiplicity of peaks is denoted as broad singlet (bs), singlet (s), doublet (d), triplet (t), quadruplet (q), and multiplet (m). Coupling constants *J* are given in Hertz. IR spectra were recorded in the solid state at a resolution of 2 cm^{-1} on a Bruker ALPHA-P Diamant ATR-FTIR. HPLC-MS was carried out on an Agilent Technologies 1260 Infinity II machine on a reverse phase C18 column. Gradient elution was carried out with acetonitrile and water with 0.1 % formic acid added. A linear gradient from 30 % MeCN to 100 % MeCN over 20 min was maintained with a flow rate of 0.8 mL/min. Samples were ionized by electrospray ionization and detected by mass spectrometer.

Elemental analyses (C, H, N) were carried out by the Department of Inorganic Chemistry at the Graz University of Technology (Heraeus Vario Elementar automatic analyzer).

2 Syntheses

[$WBr_2(CO)_3(pzH)_2$] (**1**). [$WBr_2(CO)_3(MeCN)_2$] (700 mg, 1.37 mmol) was dissolved in CH_2Cl_2 (8 mL), and 3,5-dimethyl pyrazole (265 mg, 2.76 mmol) was added and the reaction was stirred at room temperature for 15 min. The product mixture was filtered through a pad of Celite, the filtrate was reduced to dryness *in vacuo* and the resulting solids were recrystallized from CH_2Cl_2/n-heptane at -30 °C to afford 715 mg (84 %) of the product as orange crystals.

NMR: δ_H (300 MHz, $CDCl_3$) 11.15 (s, 2H, N-H), 5.96 (d, *J*=2.4 Hz, 2H, pyrazole-CH), 2.262 (s, 6H, CH_3), 2.255 (s, 6H, CH_3) ppm. δ_C (75 MHz, $CDCl_3$) 228.4 (CO), 154.9 (pz-C), 143.3 (pz-C), 108.3 (pz-C), 15.6 (CH_3), 11.2 (CH_3) ppm.

IR: ν_{max}/cm^{-1} 3392, 3340, 2023 (CO), 1939 (CO), 1920 (CO), 1885 (CO).

Elemental Analysis: Found: C, 24.95; H, 2.60; N, 9.17. $C_{13}H_{16}Br_2N_4O_3W$ requires: C, 25.19; H, 2.60; N, 9.04.

[WBr_2(pz-$NHCCH_3$)$(CO)_3$] (**1i**). A solution of **1** (205 mg, 330 µmol) in acetonitrile (1.5 mL) was prepared and left standing at room temperature for 12 h. The deep red solution was then stored at -30 °C upon which the product crystallized from the solution. The supernatant was decanted off and the orange solids were washed with diethyl ether (2 mL) and dried *in vacuo* to give 21 mg (10 %) of product.

NMR: δ_H (300 MHz, CD_3CN) 10.30 (s, 1H, pz-N**H**CMe), 6.42 (s, 1H, pz-CH), 2.81 (d, *J*=1.1, 3H, NHCC**H**$_3$), 2.62 (d, *J*=0.9, 3H, pz-CH_3), 2.51 (s, 3H, pz-CH_3) ppm.

IR: ν_{max}/cm^{-1} 3280, 3096, 2033 (CO), 1962 (CO), 1899 (CO).

[$WBr_2(C_2H_2)(pzH)_2(CO)$] (**2**) and **[$WBr_2(C_2D_2)(pzH)_2(CO)$]** (**2D**). The 1:1 mixture of [$WBr_2(C_2H_2)(MeCN)_2(CO)$]/[$WBr_2(C_2H_2)_2(MeCN)(CO)$] (865 mg, 1.8 mmol W) and pzH (370 mg, 3.75 mmol) were placed into a Schlenk flask and CH_2Cl_2 (10 mL) was added. After stirring at room temperature for 3 h, all volatiles were removed *in vacuo*. Acetonitrile (2 mL) was added to the dry residue and the mixture was homogenized by stirring before adding the suspension onto a Celite pad. The liquid fraction was then eluted and the residual insoluble product was washed with acetonitrile (3 x 2 mL) times. The acetonitrile filtrate was discarded before eluting

the desired product with CH_2Cl_2 (20 mL). The combined CH_2Cl_2 filtrates were reduced to dryness *in vacuo* to afford 410 mg (38 %) of a bright blue fine powder. The use of the precursor with deuterium labelled acetylene [$WBr_2(C_2\mathbf{D}_2)(MeCN)_2(CO)$]/ [$WBr_2(C_2\mathbf{D}_2)_2(MeCN)(CO)$] allowed the preparation of [$WBr_2(C_2\mathbf{D}_2)(pzH)_2(CO)$] (**2D**) by the same procedure.

NMR: δ_H (300 MHz, $CDCl_3$) 12.98 (s, 2H, C_2H_2), 11.90 (s, 2H, pz-NH), 5.77 (d, *J*=2.5 Hz, 2H, pz-CH), 2.33 (s, 6H, pz-CH_3), 2.22 (s, 6H, pz-CH_3) ppm. δ_C (75 MHz, $CDCl_3$) 229.5 (CO), 156.1 (**C**-CH_3), 142.1 (**C**-CH_3), 107.3 (pz-**C**H), 16.9 (pz-**C**H_3), 10.9 (pz-**C**H_3) ppm.

IR: ν_{max}/cm^{-1} 1913 (CO) cm^{-1}.

Elemental Analysis: Found: C, 26.59; H, 3.00; N, 9.14. $C_{13}H_{18}Br_2N_4OW$ requires: C, 26.47; H, 3.08; N, 9.50.

[$WBr(C_2H_2)(pzH)(pz\text{-}NHCCH_3)(CO)$]Br (**2i**) and **[$WBr(C_2D_2)(pzH)(pz\text{-}NHCCH_3)(CO)$]Br** (**2iD**). A solution of **2** (121 mg, 205 µmol) was stirred in acetonitrile (5 mL) for 24 h. All volatiles were removed *in vacuo* to afford the product in quantitative yield (125 mg, 98 %) as a blue powder. Crystals suitable for X-ray diffraction analysis were obtained from MeCN/Et_2O at -30 °C. The use of [$WBr_2(C_2\mathbf{D}_2)(pzH)_2(CO)$] (**2D**) gave complex [$WBr(C_2\mathbf{D}_2)(pzH)(pz\text{-}NHCCH_3)(CO)$]Br (**2iD**) by the same procedure.

NMR: δ_H (300 MHz, $CDCl_3$) 13.33 (s, 1H, C_2H_2), 13.28 (s, 1H, NH), 12.96 (s, 1H, NH), 12.86 (s, 1H, C_2H_2) 6.15 (s, 1H, pz-CH), 5.81 (s, 1H, pz-CH), 3.17 (s, 3H, NC-CH_3), 2.60 (s, 4H, pz-CH_3), 2.57 (s, 4H, pz-CH_3), 2.46 (s, 3H, pz-CH_3), 2.32 (s, 3H, pz-CH_3) ppm. δ_H (300 MHz, CD_3CN) 13.55 (s, 1H), 13.21 (s, 1H), 12.96 (s, 1H), 12.68 (s, 1H), 6.28 (s, 1H), 5.94 (s, 1H), 3.10 (d, *J*=1.2, 3H), 2.55 (s, 4H), 2.51 (s, 3H), 2.40 (s, 3H), 2.27 (s, 3H) ppm. δ_C (75 MHz, $CDCl_3$) 229.9 (t, J^{WC} = 71.7 Hz, CO), 205.4 (C_2H_2), 199.7 (C_2H_2), 162.8, 159.2, 158.3, 146.0, 145.9, 114.5, 107.7, 22.0 (CH_3), 17.8 (CH_3), 17.6 (CH_3), 15.0 (CH_3), 10.8 (CH_3) ppm.

IR: ν_{max}/cm^{-1} 3036 (NH), 1926 (CO).

Elemental Analysis: Found: C, 29.14; H, 3.40; N, 10.96. $C_{15}H_{21}Br_2N_5OW\cdot 0.05\ CH_3CN$ requires: C, 28.65; H, 3.37; N, 11.17.

[$W(C_2H_2)(CO)(pzH)(\mu\text{-}OH)_2(\mu\text{-}pz)W(C_2H_2)(CO)(pzH)$]Br (**3**). In a screw-cap vial, **2** (40 mg) was dissolved in CH_2Cl_2 (2 mL). The solution was layered with aqueous NaOH (3.75 M, 40 µL, 2.2 equiv) and left standing under N_2 atmosphere for 5 days. During this time needle-like

crystals had formed. The supernatant was removed by cannula and the crystals were washed with CH_2Cl_2 (2 x 0.5 mL) and water (2 x 2 mL). Drying the product *in vacuo* afforded 10 mg (30 %) of violet, crystalline needles.

NMR: δ_H (300 MHz, $(CD_3)_2SO$) 13.50 (s, 2H, C_2H_2), 12.40 (s, 2H, C_2H_2), 11.62 (s, 2H, NH), 8.84 (s, 2H, OH), 5.88 (s, 2H, pz-CH), 5.67 (s, 1H, μ-pz-CH), 2.30 (s, 6H, CH_3), 1.96 (s, 6H, CH_3), 1.67 (s, 6H, CH_3) ppm. δ_C (75 MHz, $(CD_3)_2SO$) 238.7 (CO), 205.9 (C_2H_2), 196.1 (C_2H_2), 153.2 (pz-C), 147.1 (pz-C), 142.7 (pz-C), 105.9 (pz-C), 54.9 (pz-C), 15.8 (pz-CH_3), 14.9 (pz-CH_3), 10.1 (pz-CH_3) ppm.

IR: ν_{max}/cm^{-1} 3212 (ν_{OH}), 1896 (CO).

Elemental Analysis: Found: C, 28.34; H, 3.05; N, 8.84. $C_{21}H_{29}BrN_6O_4W_2 \cdot 0.5\ CH_2Cl_2$ requires: C, 28.08; H, 3.29; N, 9.14.

3 NMR spectra of compounds

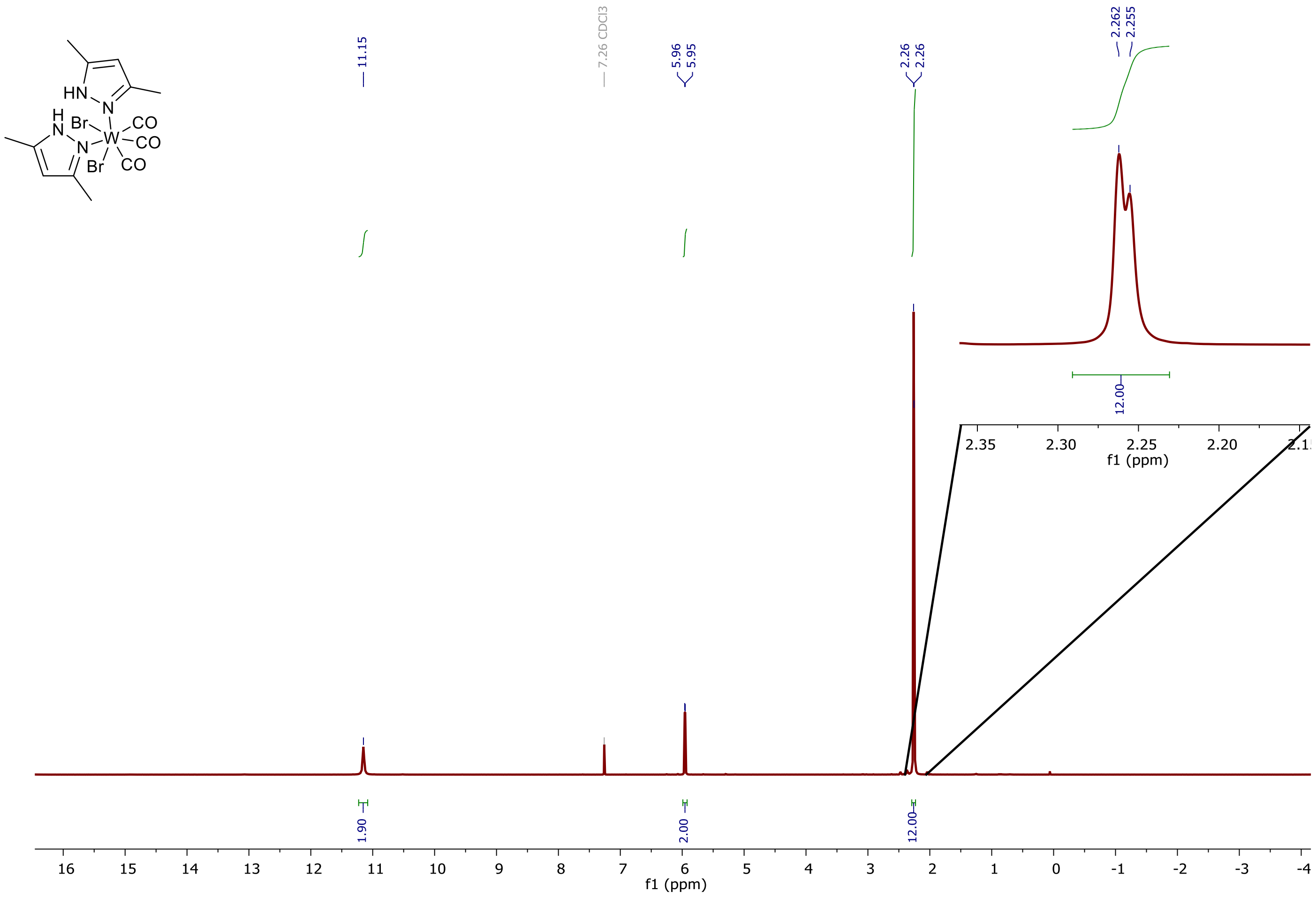

Fig. S1. ^{1}H NMR spectrum of [$WBr_2(pzH)_2(CO)_3$] (**1**) in $CDCl_3$ with expanded view of the signals corresponding to the methyl groups from 2.15-2.35 ppm.

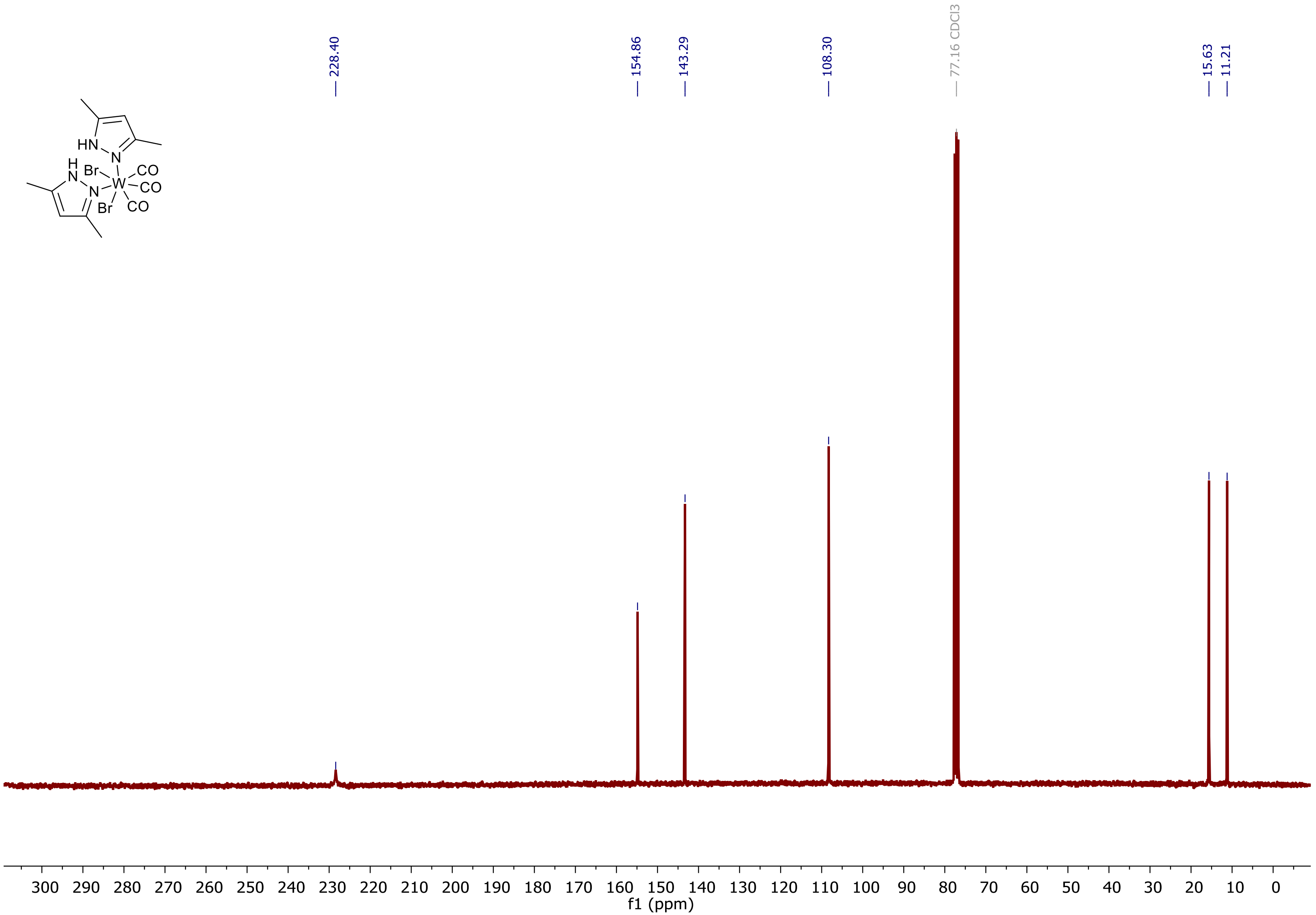

Fig. S2. ^{13}C NMR spectrum of $[WBr_2(pzH)_2(CO)_3]$ (**1**) in $CDCl_3$.

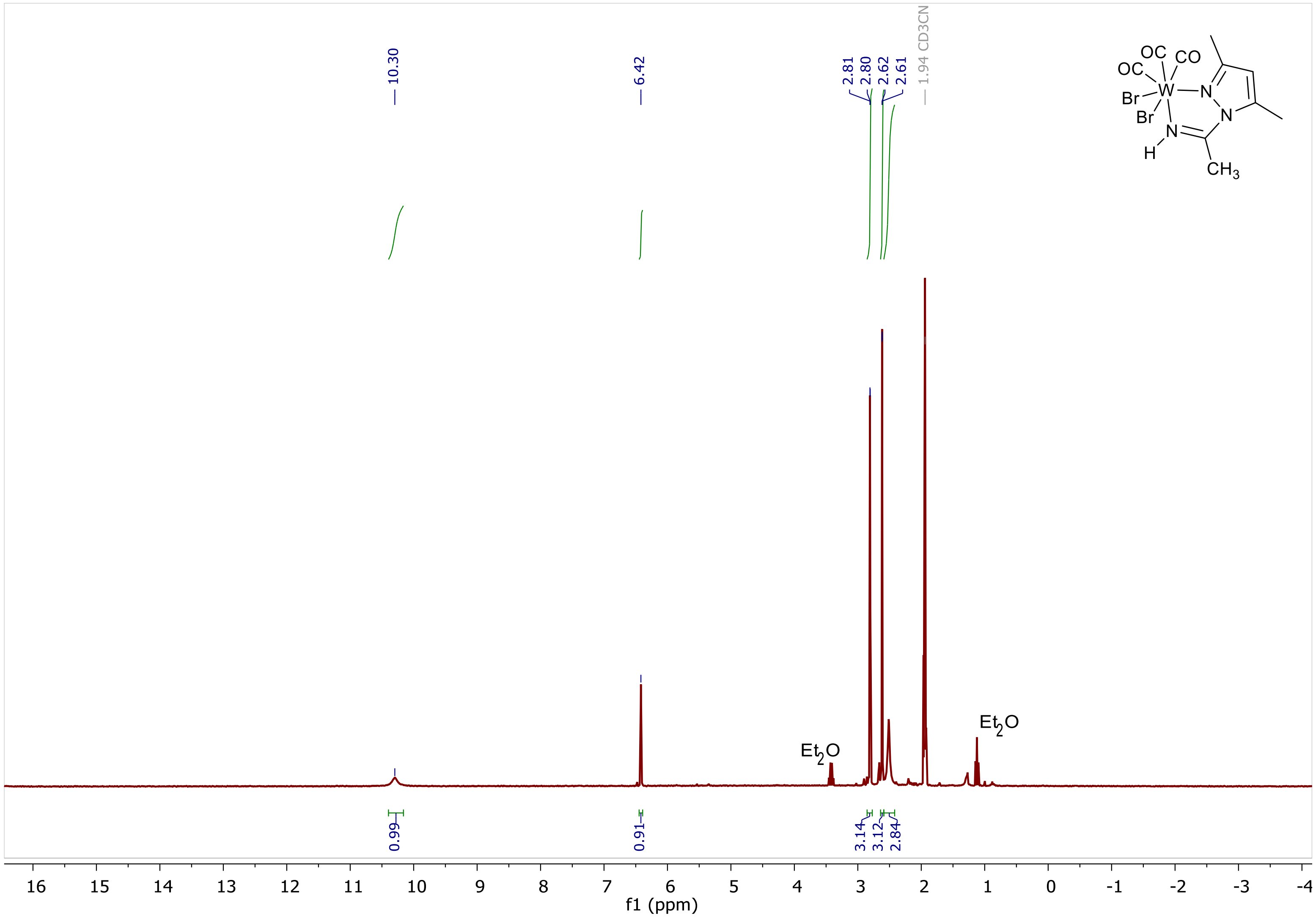

Fig. S3. ^{1}H NMR spectrum of $[WBr_2(pz\text{-}NHCCH_3)(CO)_3]$ (**1i**) in CD_3CN.

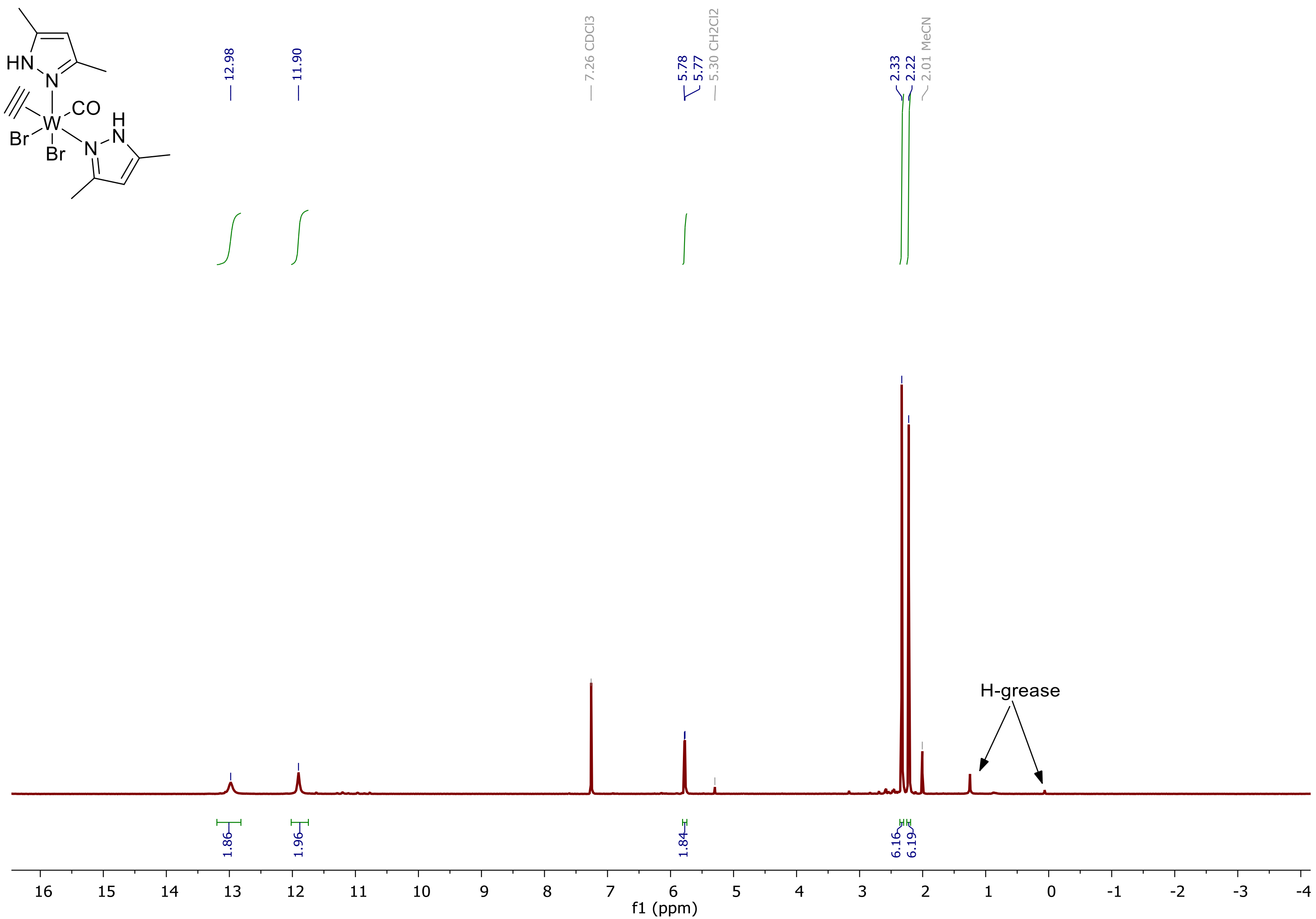

Fig. S4. ^{1}H NMR spectrum of $[WBr_2(C_2H_2)(pzH)_2(CO)]$ (**2**) in $CDCl_3$.

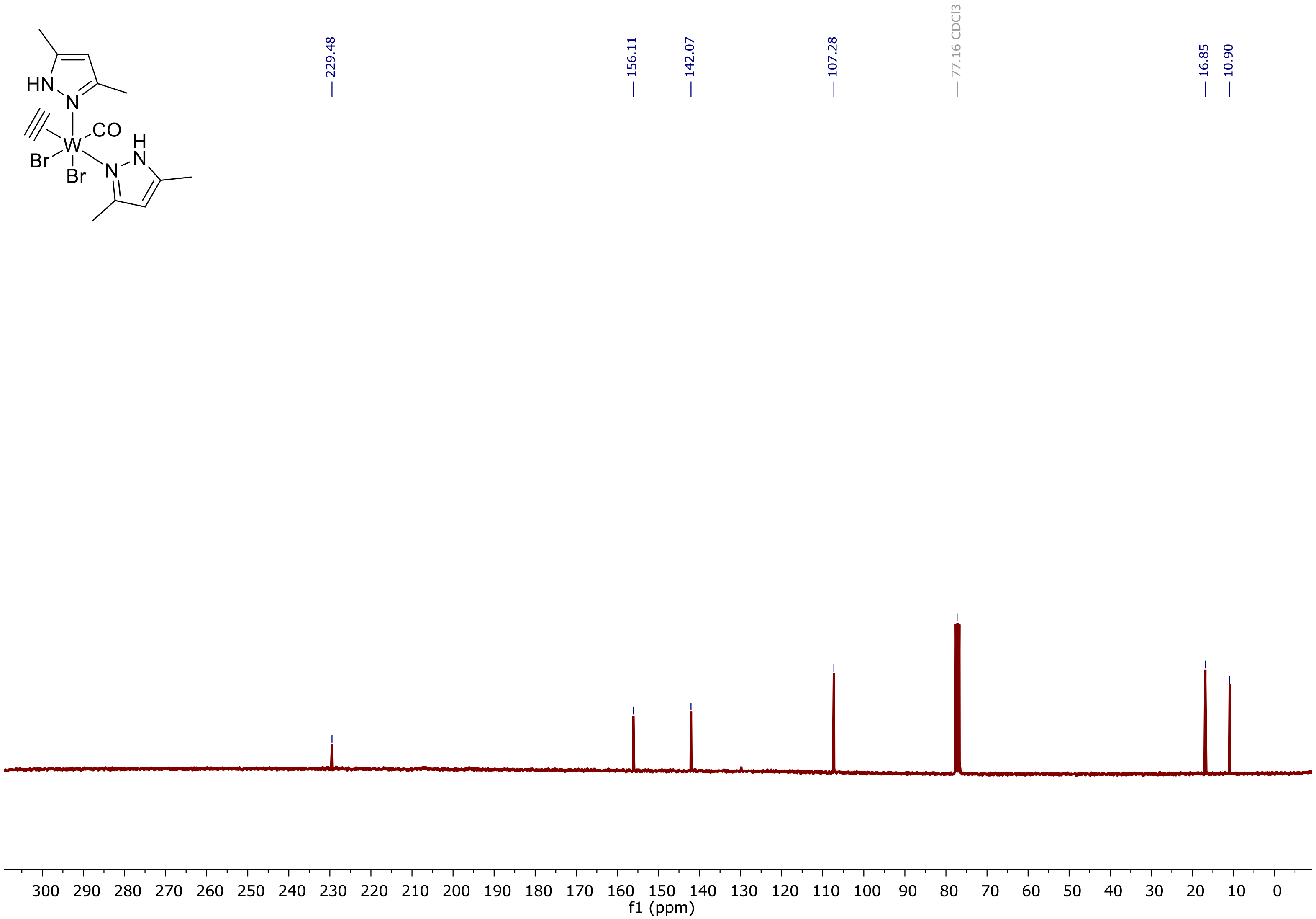

Fig. S5. ^{13}C NMR spectrum of $[WBr_2(C_2H_2)(pzH)_2(CO)]$ (**2**) in $CDCl_3$.

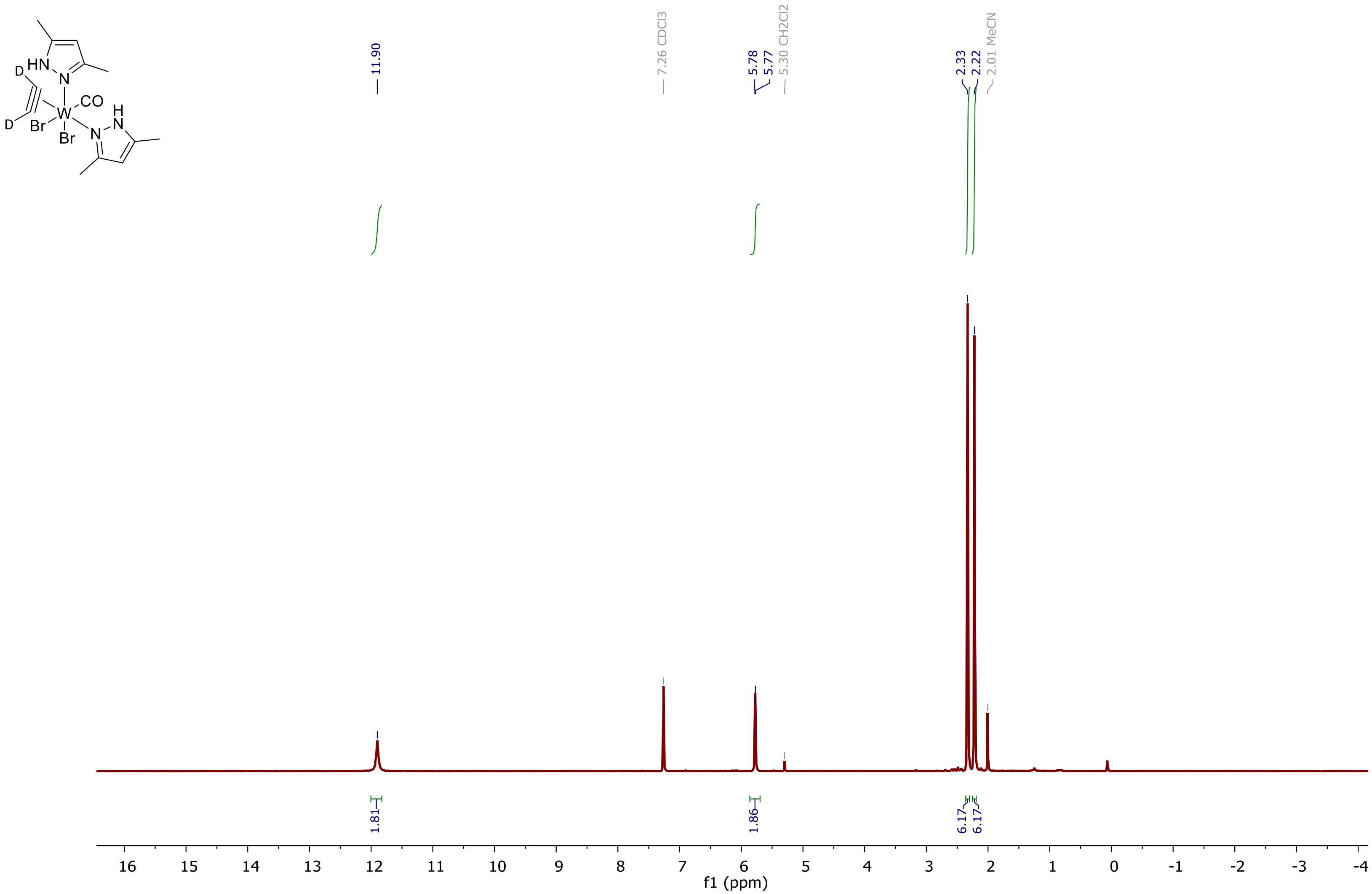

Fig. S6. ^{1}H NMR spectrum of $[WBr_2(C_2D_2)(pzH)_2(CO)]$ (**2D**) in $CDCl_3$.

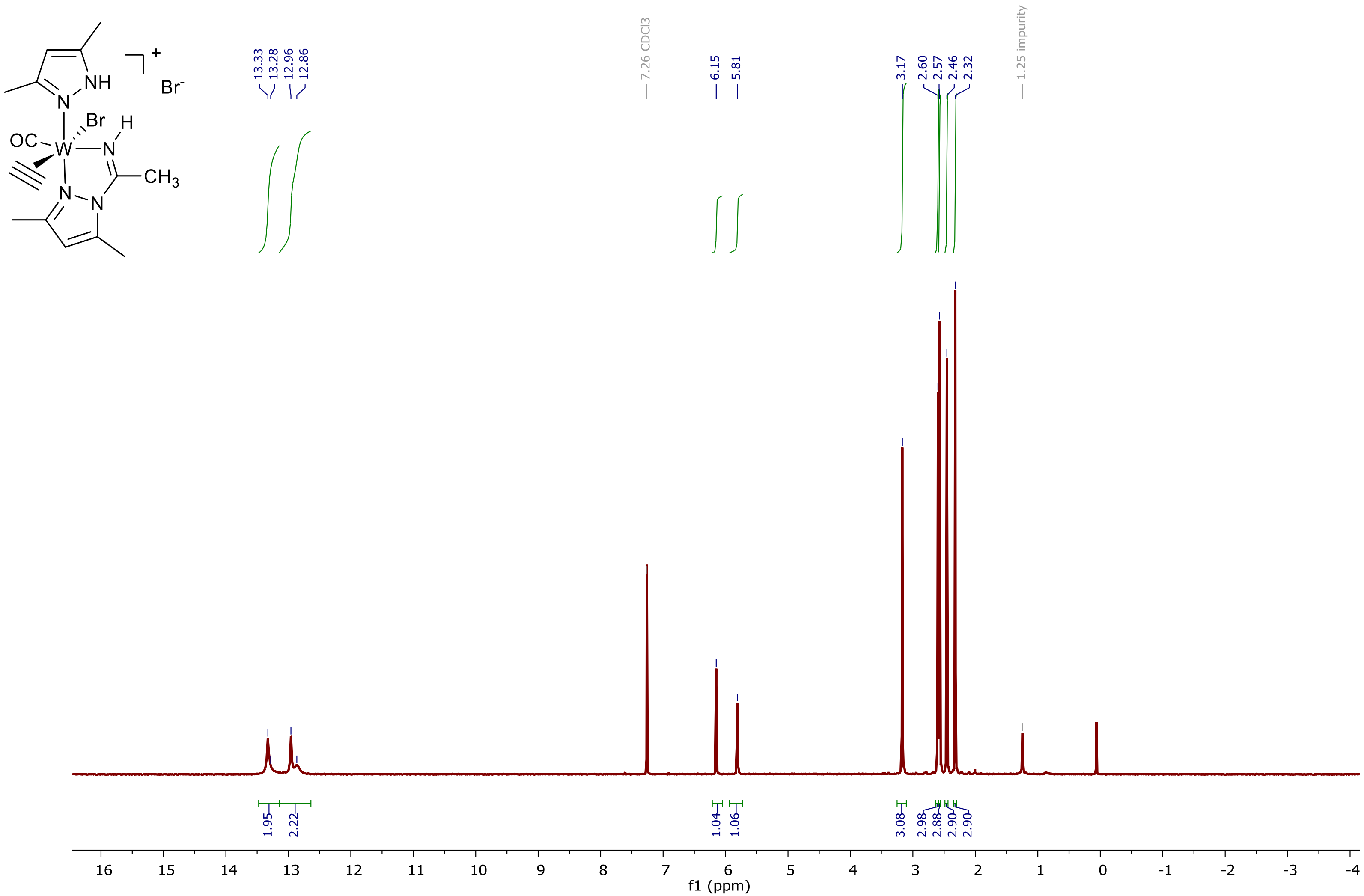

Fig. S7. ^{1}H NMR spectrum of $[WBr(C_2H_2)(pzH)(pz\text{-}NHCCH_3)(CO)]Br$ (**2i**) in $CDCl_3$.

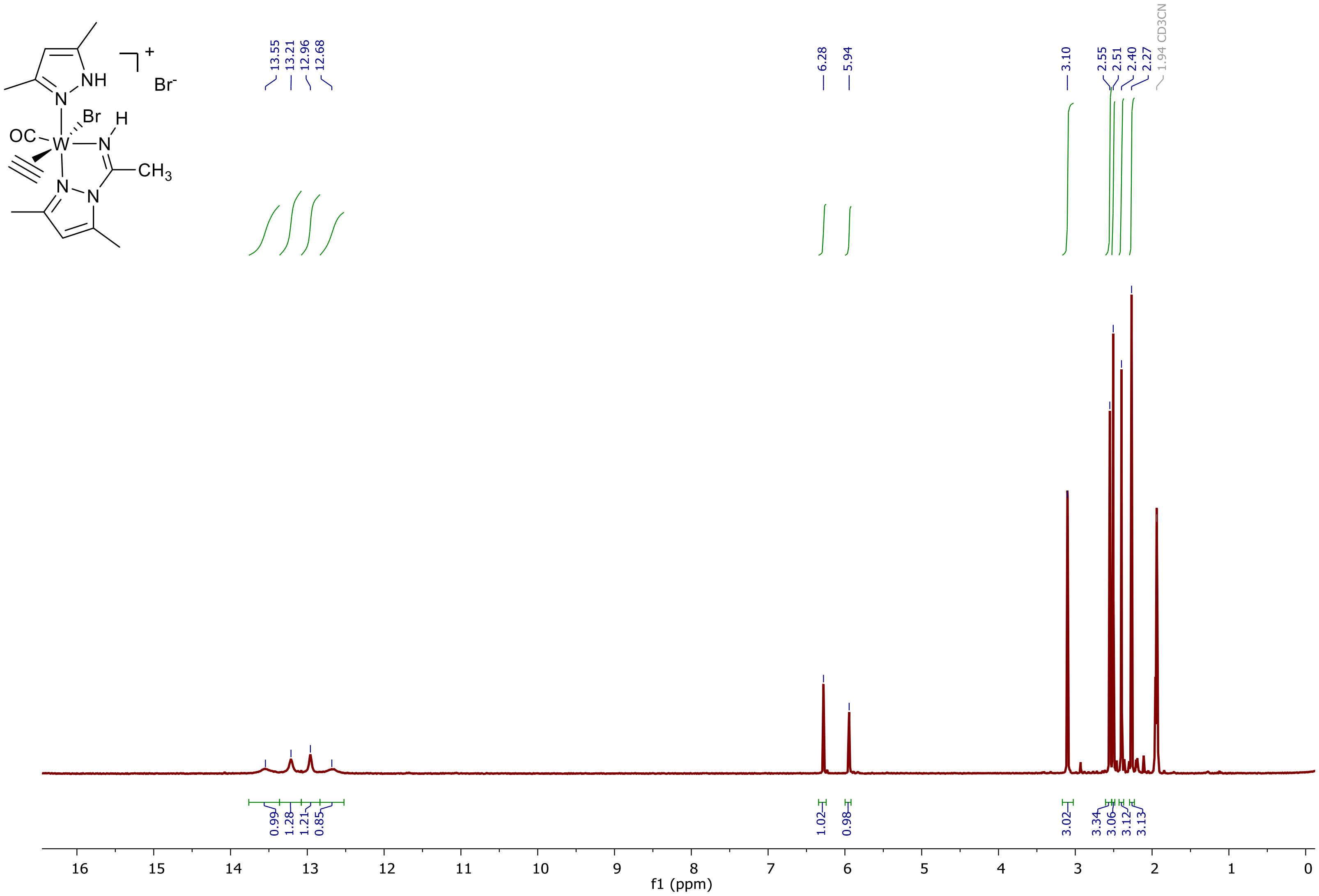

Fig. S8. ^{1}H NMR spectrum of $[WBr(C_2H_2)(pzH)(pz\text{-}NHCCH_3)(CO)]Br$ (**2i**) in CD_3CN.

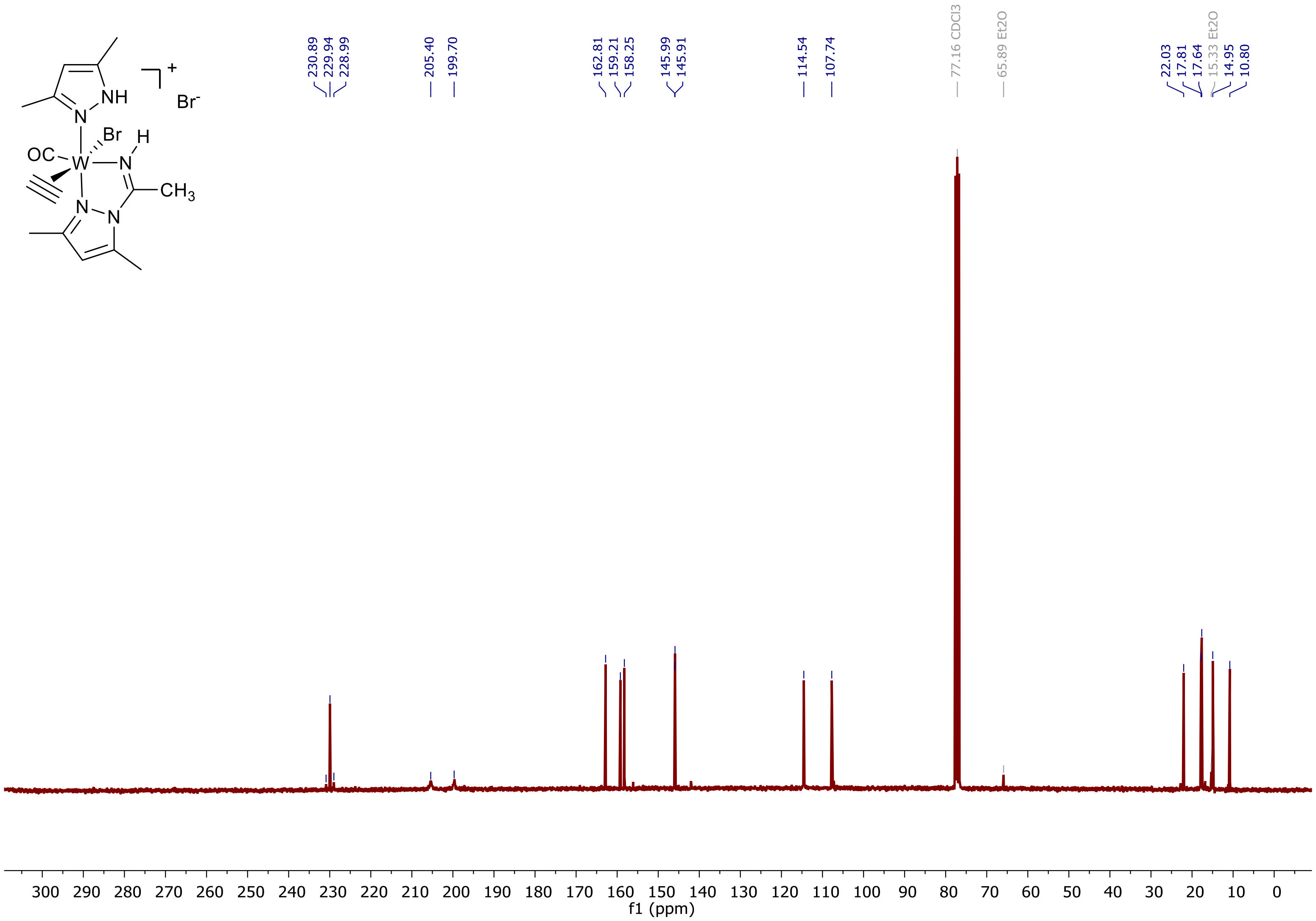

Fig. S9. ^{13}C NMR spectrum of $[WBr(C_2H_2)(pzH)(pz\text{-}NHCCH_3)(CO)]Br$ (**2i**) in $CDCl_3$.

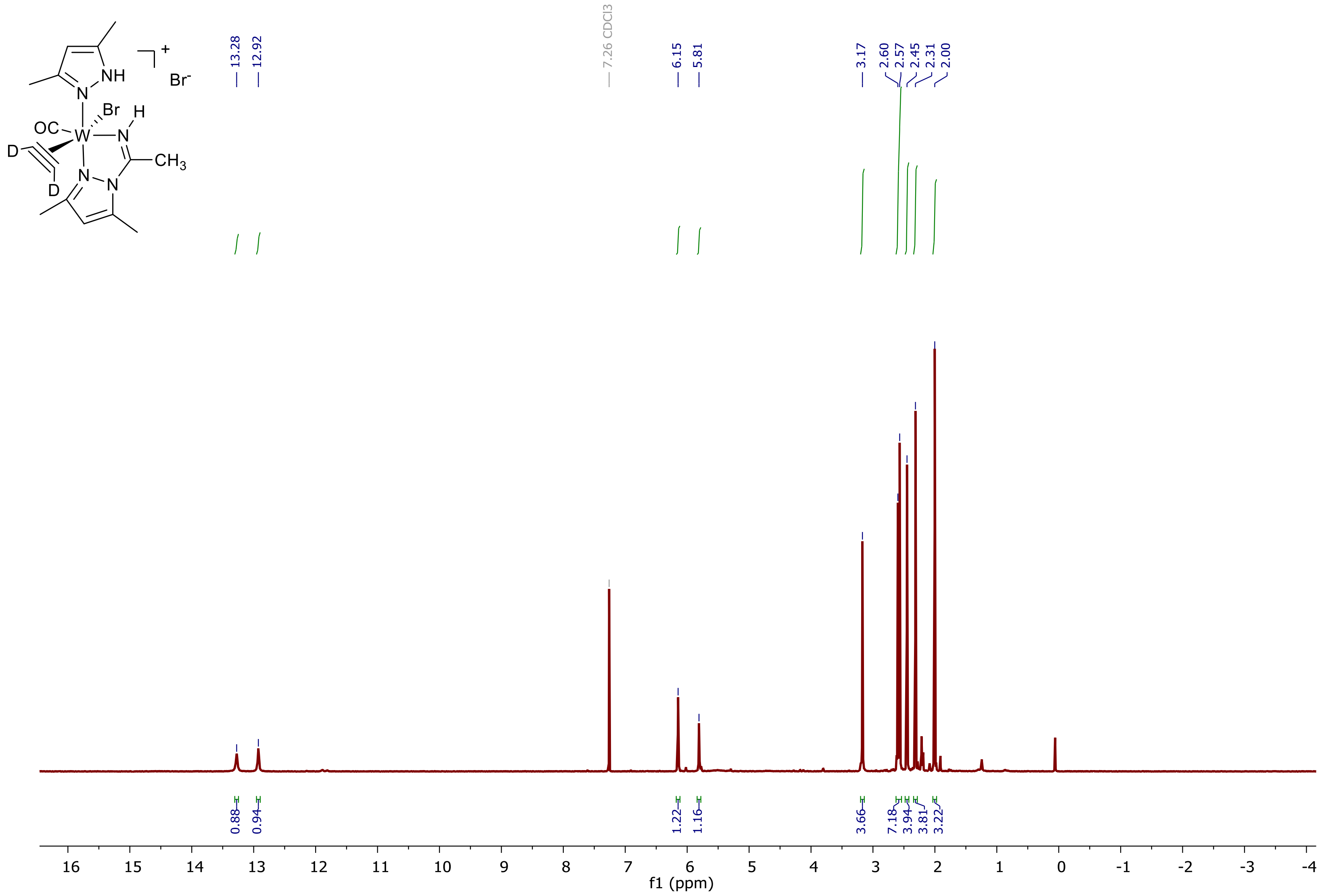

Fig. S10. ^{1}H NMR spectrum of [WBr(C_2D_2)(pzH)(pz-NHCCH$_3$)(CO)]Br (**2iD**) in $CDCl_3$.

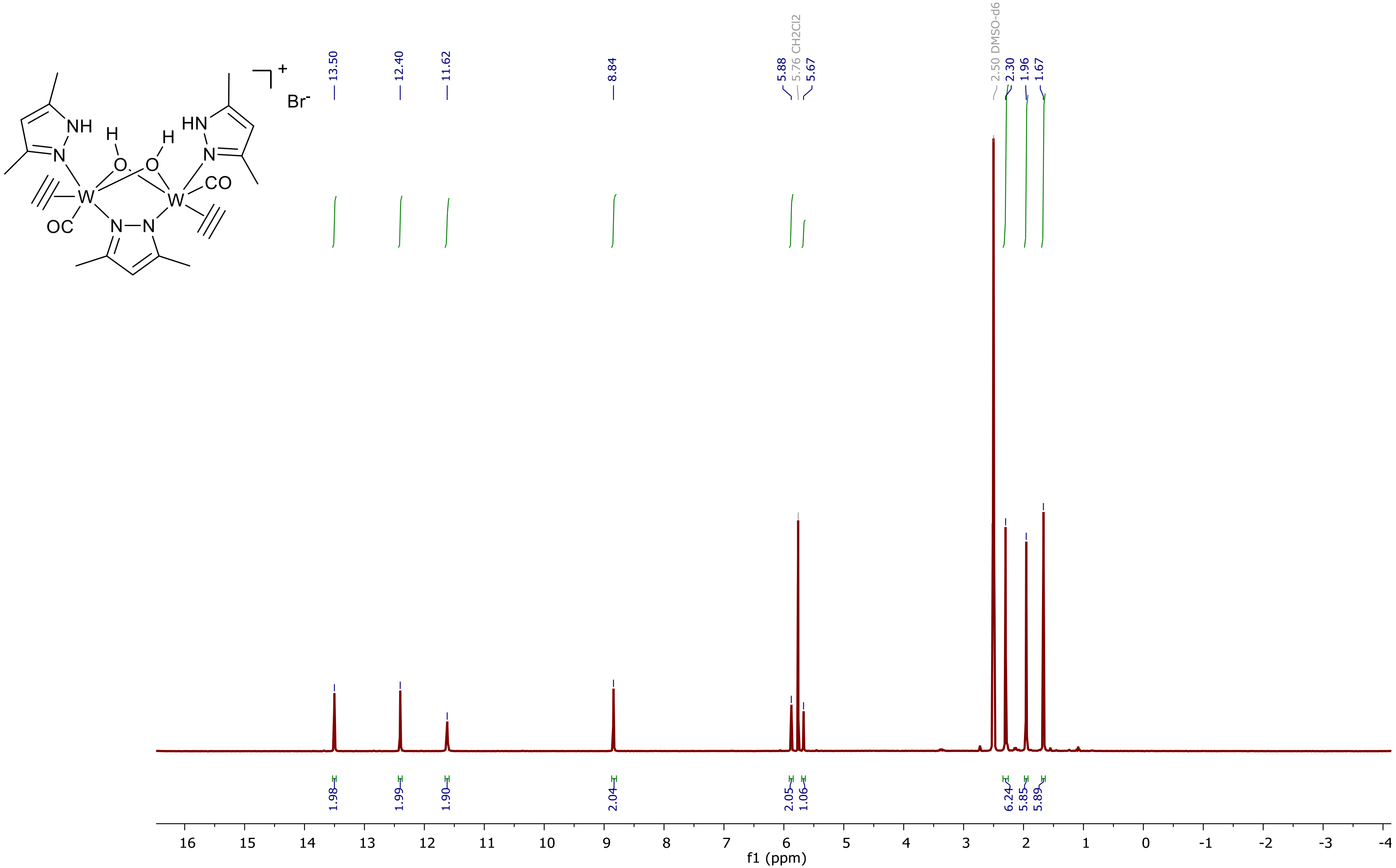

Fig. S11. ^{1}H NMR spectrum of [W(C_2H_2)(CO)(pzH)(μ-OH)$_2$(μ-pz)W(C_2H_2)(CO)(pzH)]Br (**3**) in $(CD_3)_2SO$.

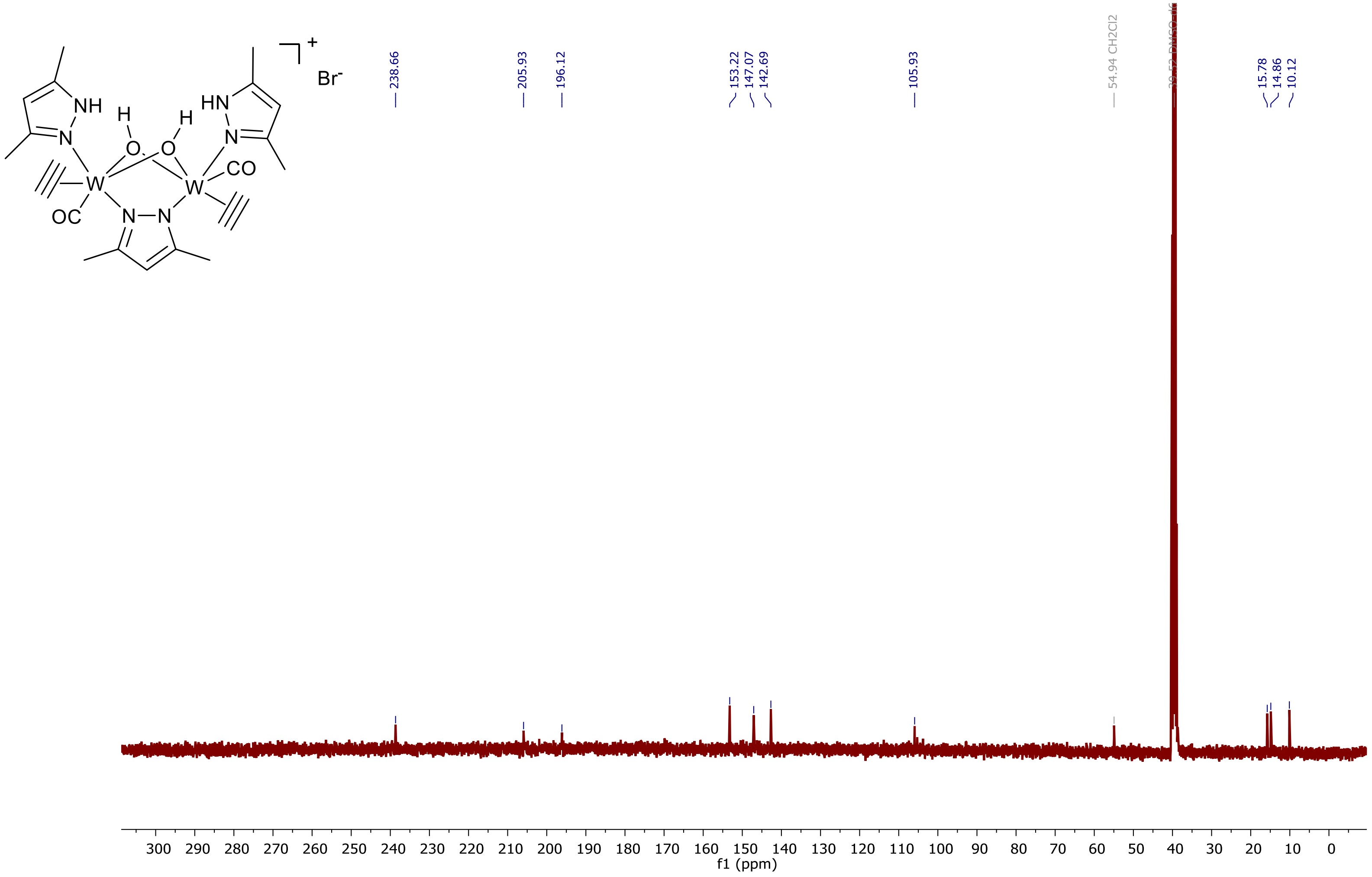

Fig. S12. ^{13}C NMR spectrum of $[W(C_2H_2)(CO)(pzH)(\mu\text{-}OH)_2(\mu\text{-}pz)W(C_2H_2)(CO)(pzH)]Br$ (**3**) in $(CD_3)_2SO$.

4 Reactivity studies

Formation of aldehyde over time

A stock solution of **1** (105 mg, 169 µmol) and mesitylene (9.47 µmol, 56 µmol) as an internal standard in CD_3CN (5950 µL; 7 x 850 µL) was prepared and left standing at room temperature for 12 h. Aliquots of the stock solution (850 µL each) were then placed in 25 mL Schlenk flasks, and at -10 °C (ice/NaCl) under acetylene counterflow, H_2O (2.18 µL, 121 µmol, 3 equiv.) was added quickly before closing the flask and allowing for saturation of the solution with acetylene under vigours stirring for 5 min. The sealed flasks were then placed in an oil bath preheated to 60 °C and heated for the specific time stated below in Table S1. Flasks that were removed from the oil bath were quickly cooled to 0 °C and under N_2 counterflow samples for ^{1}H NMR spectroscopy were taken directly. The ^{1}H NMR samples were stored at -30 °C prior to measurement. The amount of formed aldehyde is evaluated by comparing the relative integral of the internal mesitylene standard at 6.80 ppm with the integral of the aldehyde signals at 9.69 ppm for acetaldehyde, and at 9.46 ppm for crotonaldehyde, respectively, in the ^{1}H NMR spectrum (Fig. S17).

Table S1. Detected conversion (% vs. W) of acetaldehyde, crotonaldehyde, and *N*-vinyl-3,5-dimethyl pyrazole from reactions of **1**, H_2O (5 equiv), and excess acetylene in CD_3CN at 60 °C.

entry	t (h)	acetaldehyde (%)	crotonaldehyde (%)	*N*-vinyl-3,5-dimethyl pyrazole (%)
**1**	1	12	2	8
**2**	2	26	2	5
**3**	4	50	6	n.d.[a]

[a] The signal-to-noise ratio did not allow for quantification.

Control experiment with acetaldehyde. A stock solution of **1** (15 mg/850 µL, 24 µmol/850 µL) and mesitylene (1.12 µL/850 µL, 8 µmol/850 µL, 1/3 equiv) was prepared in CD_3CN and left standing at room temperature for 12 h. A Schlenk flask was then charged with 850 µL of this stock and under N_2-counterflow at 0 °C, acetaldehyde (2.71 µL, 48 µmol) followed by water (2.18 µmol, 121 µmol) was added. The flask was closed and placed in an oil bath preheated to 60 °C for 4 h. The sample was subsequently cooled to 0 °C and a sample for ^{1}H NMR spectroscopy was taken directly. No crotonaldehyde could be detected in the ^{1}H NMR spectrum (Fig. S21).

Formation of vinyl-pyrazole and subsequent hydrolysis. A J. Young NMR tube containing a solution of **1** (15 mg, 24 µmol), mesitylene (1.12 µL, 8 µmol), and C_2H_2 in CD_3CN (600 µL) was left standing at room temperature for 24 h before recording a ^1H NMR spectrum. The presence of *N*-vinyl-3,5-dimethyl pyrazole and the absence of aldehyde were detected. Subsequently, 1 drop of degassed H_2O was added to the mixture under inert conditions and the NMR tube was placed into an oil bath and heated to 60 °C for 24 h before recording another ^1H NMR spectrum. Acetaldehyde was detected while signals corresponding to *N*-vinyl-3,5-dimethyl pyrazole were absent from the spectrum. The stacked spectra are shown in Fig. S18.

Preparation of 1,2-dideutero acetaldehyde. C_2D_2 was prepared from calcium carbide and D_2O at 0 °C and was passed through a $CaCl_2$ column to remove residual water.

A stock solution of **1** (15 mg/850 µL, 24 µmol/850 µL) and mesitylene (1.12 µL/850 µL, 8 µmol/850 µL, 1/3 equiv) was prepared in CD_3CN and left standing at room temperature for 12 h. A Schlenk flask was then charged with 850 µL of this stock, -10 °C (ice/NaCl), and under vigorous stirring the flask was purged with C_2D_2 for 10 min before adding H_2O (2.18 µL, 121 µmol) under C_2D_2-counterflow. The flask was subsequently closed and the contents were stirred at 60 °C for 4 h. (Fig. S16). The acetaldehyde signal for the COH proton at 9.69 ppm in the ^1H NMR spectrum was notably less intense in the deuterium-labeled experiment and resonated as a triplet indicative of a vicinal -CH_2D group. The CDH_2 acetaldehyde signal resonated at 2.09 ppm as an expected triplet with the same intensity for each peak.

Derivatization aldehydes. In a Schlenk flask, a solution of **1** (60 mg, 96.7 µmol) in MeCN (1.5 mL) was cooled to -10 °C (ice/NaCl) and the solution was purged with C_2D_2 for 10 min before adding H_2O (8.7 µL, 484 µmol). The flask was closed and the mixture was heated to 60 °C for 4 h. All volatiles were condensed into a liquid-nitrogen-cooled cold trap. The derivatization of formed aldehydes with 2,4-dinitrophenyl hydrazine (DNPH) was done following a literature protocol.[3] A solution of DNPH in 1 M aqueous H_2SO_4 (1 mg/mL) was prepared. To the condensate, 5 mL of derivatization solution was added and the mixture was stirred at room temperature °C for 5 min. The derivatization products were extracted with diethyl ether (15 mL), the phases were separated and the organic phase was washed with water (5 mL) and dried over $MgSO_4$. All volatiles were removed under reduced pressure and the product mixture was analyzed by HPLC-MS. Unreacted DNPH (t_r = 4.33 min), acetaldehyde 2,4-dinitrophenyl hydrazine (r_t = 7.84 min), and crotonaldehyde 2,4-dinitrophenyl hydrazine

(r_t = 10.09 min) were detected. The corresponding *m/z* was detected for 1,2-dideutero acetaldehyde 2,4-dinitrophenyl hydrazine (AA-DNPH-*d2*), deutero acetaldehyde 2,4-dinitrophenyl hydrazine (AA-DNPH-*d*), 1,2,3,4-tetradeutero crotonaldehyde 2,4-dinitrophenyl hydrazine (CA-DNPH-*d4*), and 1,3,4-trideutero crotonaldehyde 2,4-dinitrophenyl hydrazine (CA-DNPH-*d3*) in the negative mode. Samples of commercially available acetaldehyde derivatized with DNPH employing the same protocol had an identical retention time as the AA-DNPH-*d2*. The *m/z* signals found in the negative mode clearly show deuterium-enriched AA-DNPH, however notable amounts of unlabeled AA-DNPH are detected. Prolonged derivatization times led to a decrease in deuterium-labeled AA-DNPH-products, likely as a result of proton scrambling due to imine-enamine tautomerism.

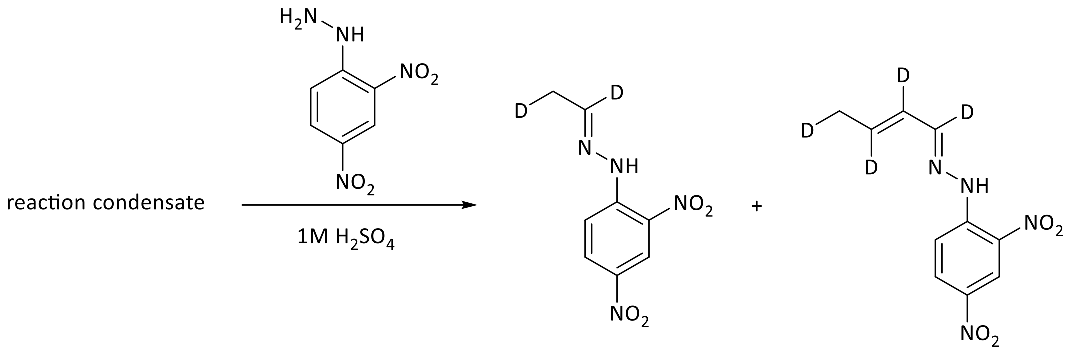

AA-DNPH-*d2*
m/z (calc): 226
found : 226, 225

CA-DNPH-*d4*
m/z (calc): 254
found: 254, 253

Scheme S1. Derivatization of aldehydes with DNPH under acidic conditions gives AA-DNPH and CA-DNPH, respectively, which were analyzed by HPLC-MS.

Hydrolysis of *N*-vinyl-3,5-dimethyl pyrazole with aqueous HCl. A solution of *N*-vinyl-3,5-dimethyl pyrazole (42 mg, 346 µmol) and mesitylene as an internal standard in CD_3CN was prepared and HCl (1.11 M, 26 µL; accounts for 0.1 equiv HCl, 5 equiv H_2O) was added. After recording a ^1H NMR spectrum, the sample was heated to 60 °C for 4 h before recording another ^1H NMR spectrum which revealed 80 % conversion of *N*-vinyl-3,5-dimethyl pyrazole to acetaldehyde (52 %) and 1,1-bis(3,5-dimethyl-1-pyrazolyl)ethane (21 %) (**Fig. S19**).

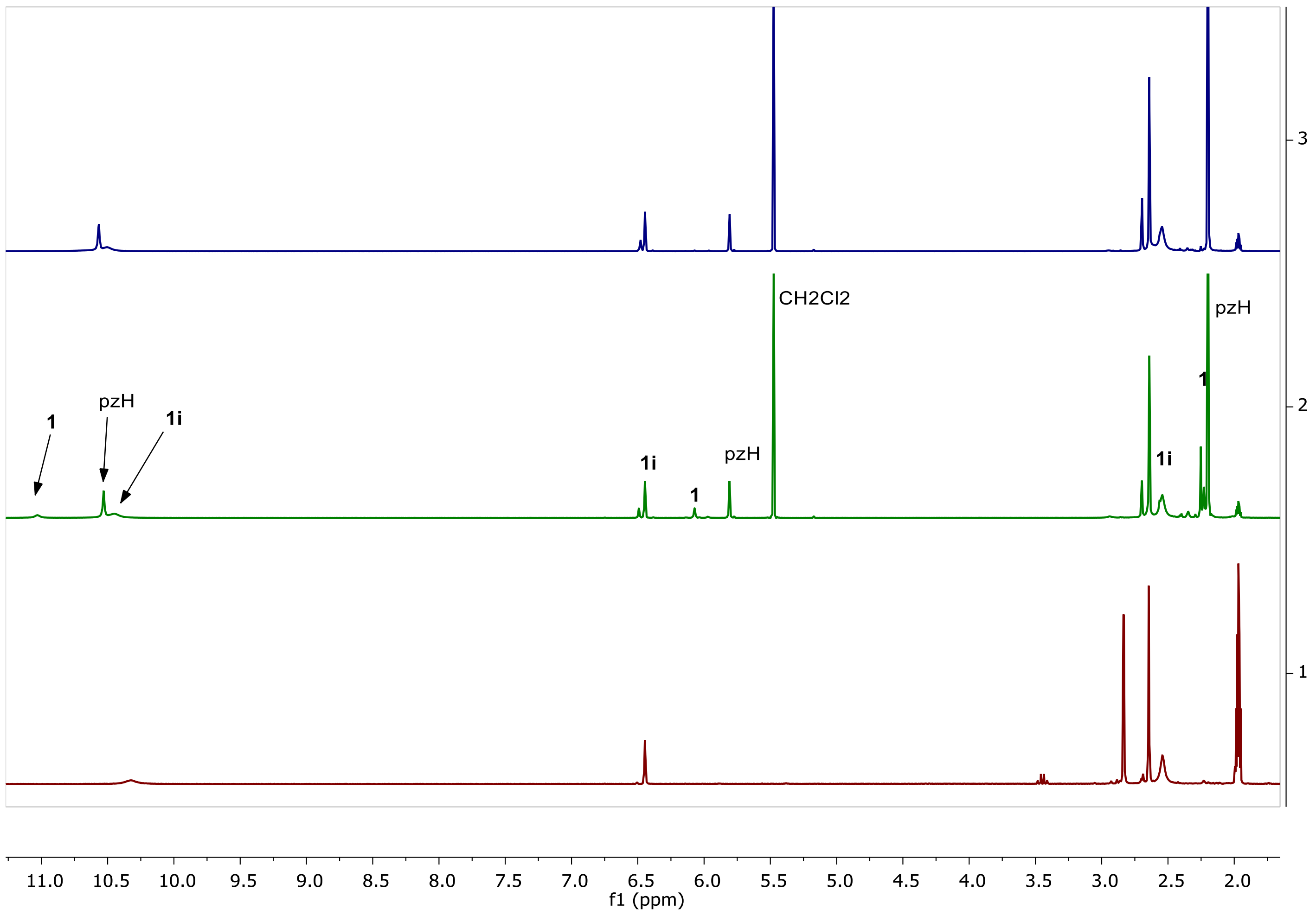

Fig. S13. Stacked ^{1}H NMR spectra of (bottom) isolated [WBr_2(pz-$NHCCH_3$)$(CO)_3$] (**1i**) in CD_3CN, (middle) [$WBr_2(pzH)_2(CO)_3$] (**1**) in CD_3CN after 2.5 h, and (top) [$WBr_2(pzH)_2(CO)_3$] (**1**) in CD_3CN after 4 h.

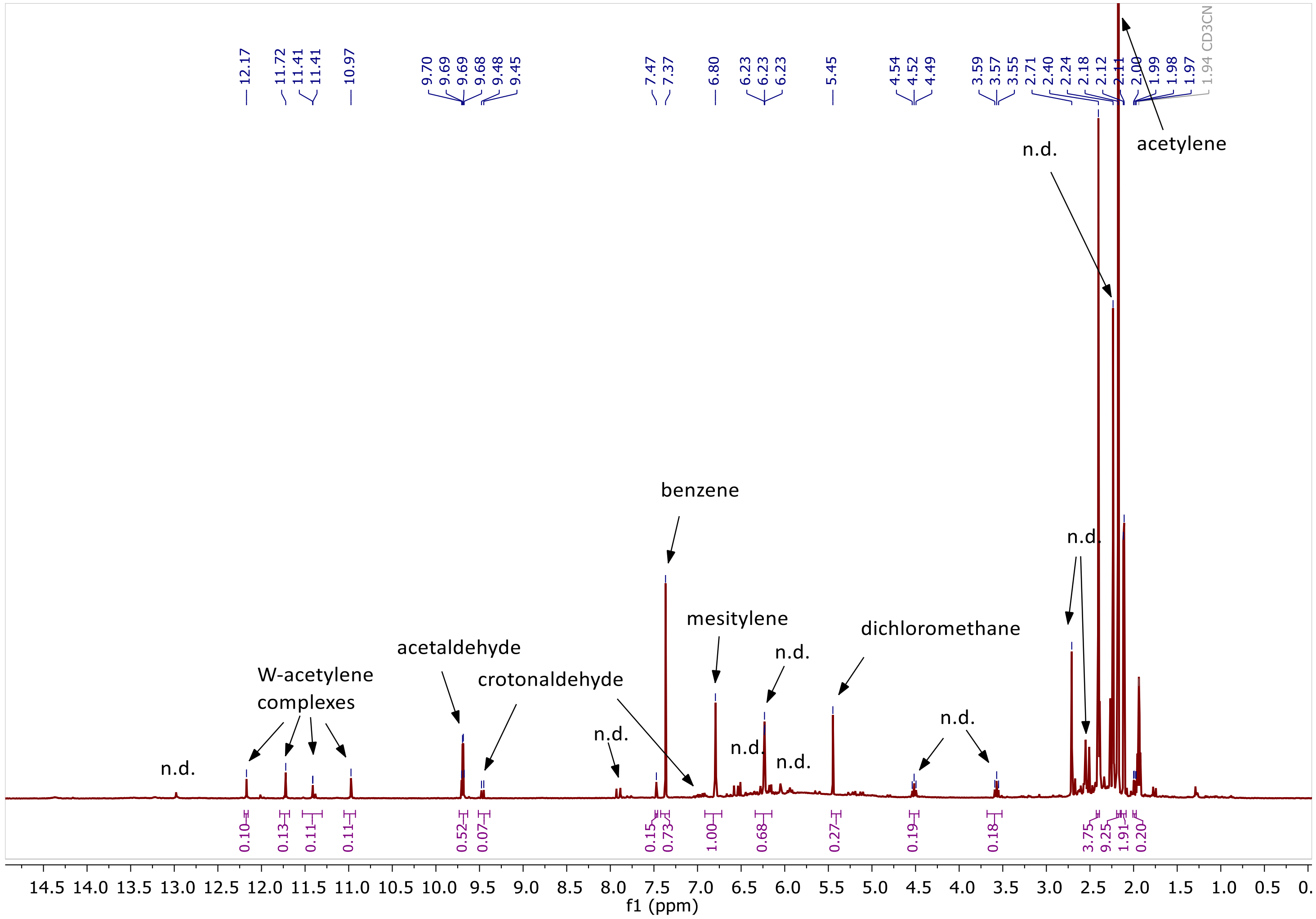

Fig. S14. ^{1}H NMR spectrum of the product mixture of a reaction of {**1i**+pzH} (prepared *in situ*), acetylene, H_2O (5 equiv), and mesitylene (1/3 equiv) in CD_3CN heated to 60 °C for 4 h. Integral of the mesitylene signal at 6.80 ppm is set to 1.00. Peaks are assigned where possible. Unknown signals are assigned the n.d. label.

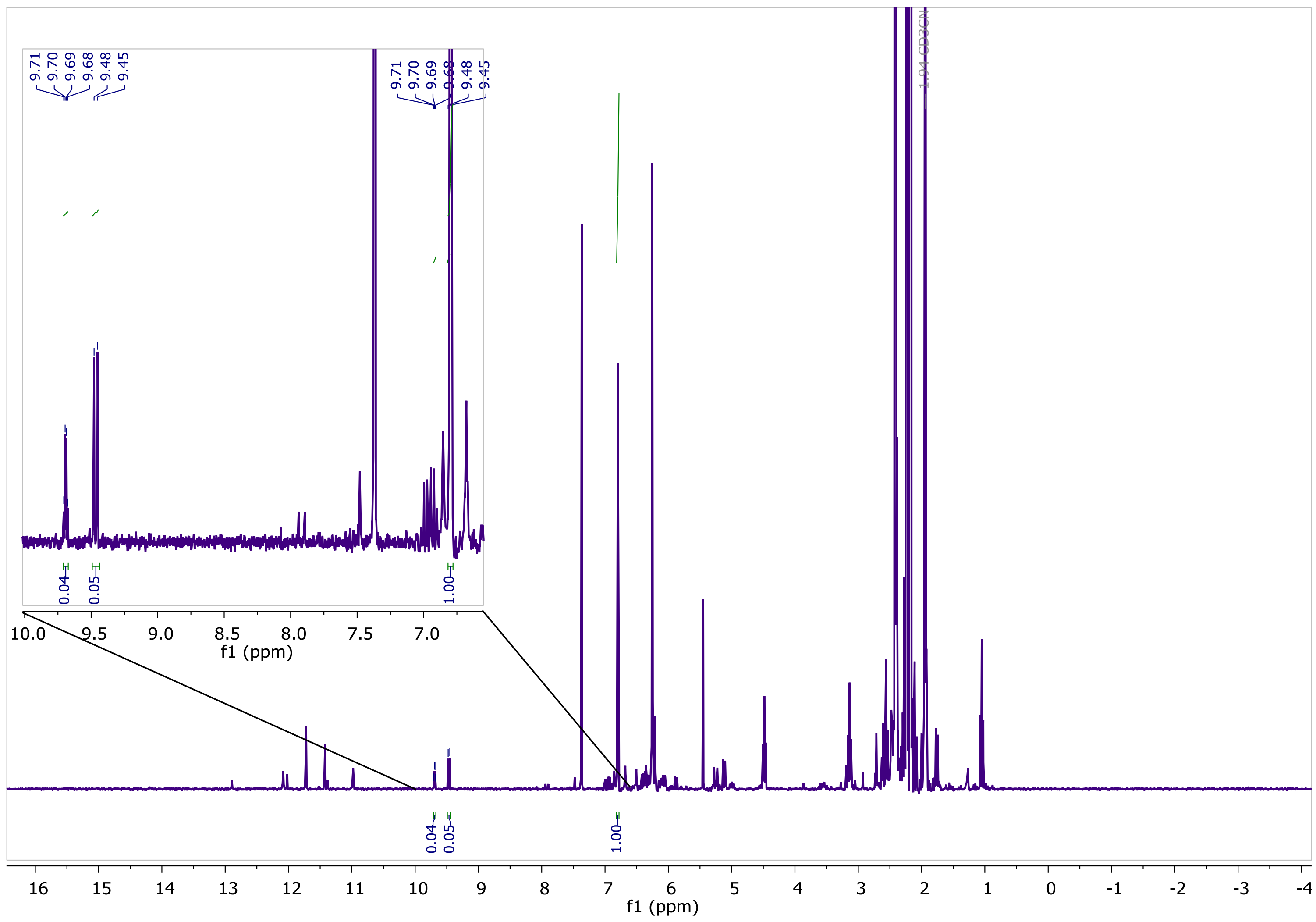

Fig. S15. ^{1}H NMR spectrum of a reaction of $[WBr_2(pzH)_2(CO)_3]$ (**1**), acetylene, H_2O (5 equiv), and mesitylene (1/3 equiv, internal standard) in CD_3CN heated to 60 °C for 4 h without waiting for the conversion of **1** to {**1i**+pzH}.

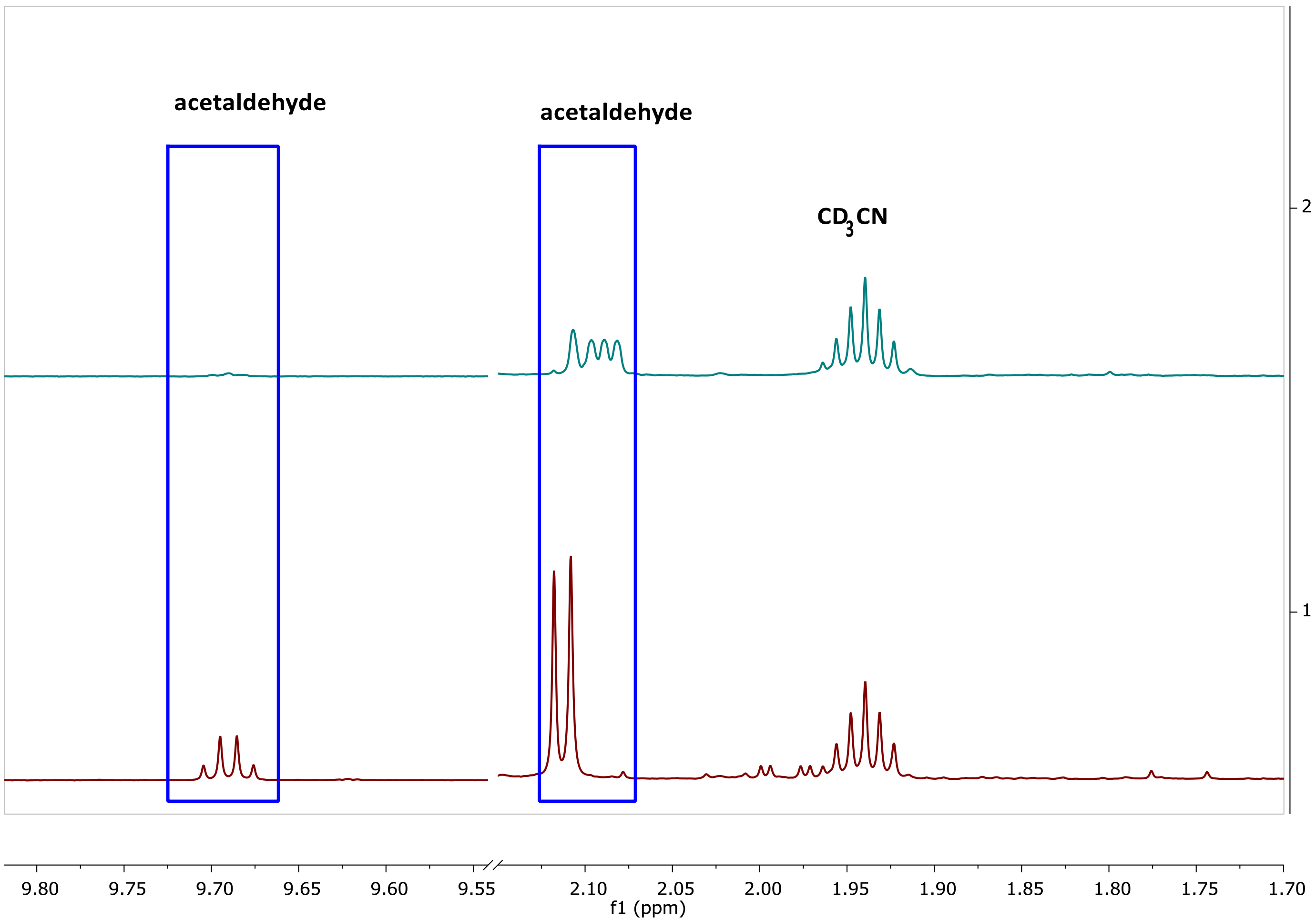

Fig. S16. Stacked ^{1}H NMR spectra of the selected signal range for acetaldehyde from a reaction of {**1i**+pzH} (prepared *in situ*) + C_2D_2 + H_2O (top), and {**1i**+pzH} (prepared *in situ*) + C_2H_2 + H_2O (bottom) in CD_3CN.

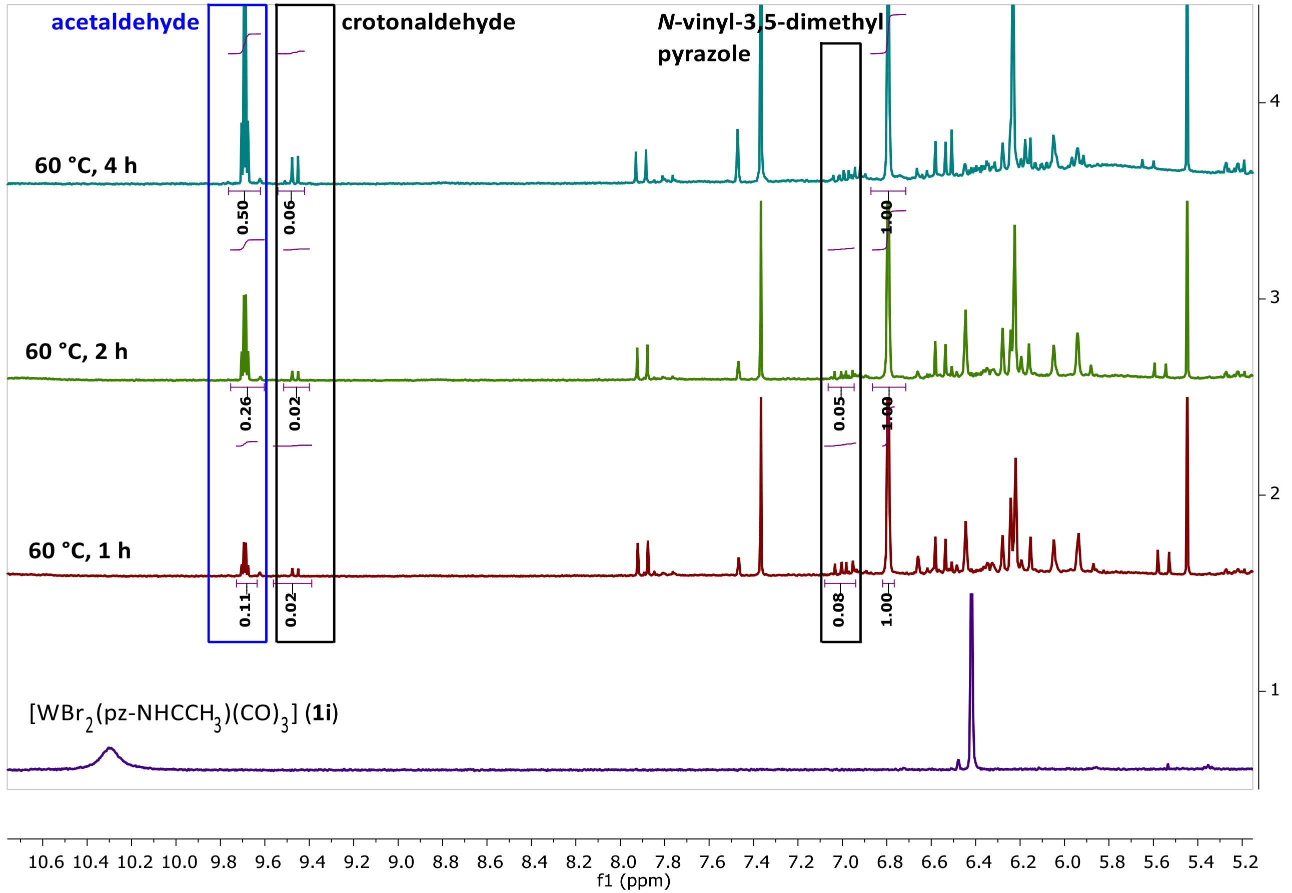

Fig. S17. Stacked ^{1}H NMR spectra of isolated $[WBr_2(pz\text{-}NHCCH_3)(CO)_3]$ (**1i**) (bottom), and the subsequent reaction of {**1i**+pzH} (prepared *in situ*) + C_2H_2 + H_2O (5 equiv) + mesitylene (internal standard, 1/3 equiv) in CD_3CN after 1, 2, and 4 h at 60 °C. Selected shifts for acetaldehyde, crotonaldehyde, and *N*-vinly-3,5-dimethyl pyrazole are highlighted. The signal of internal standard mesitylene at 6.79 ppm is set to 1.00.

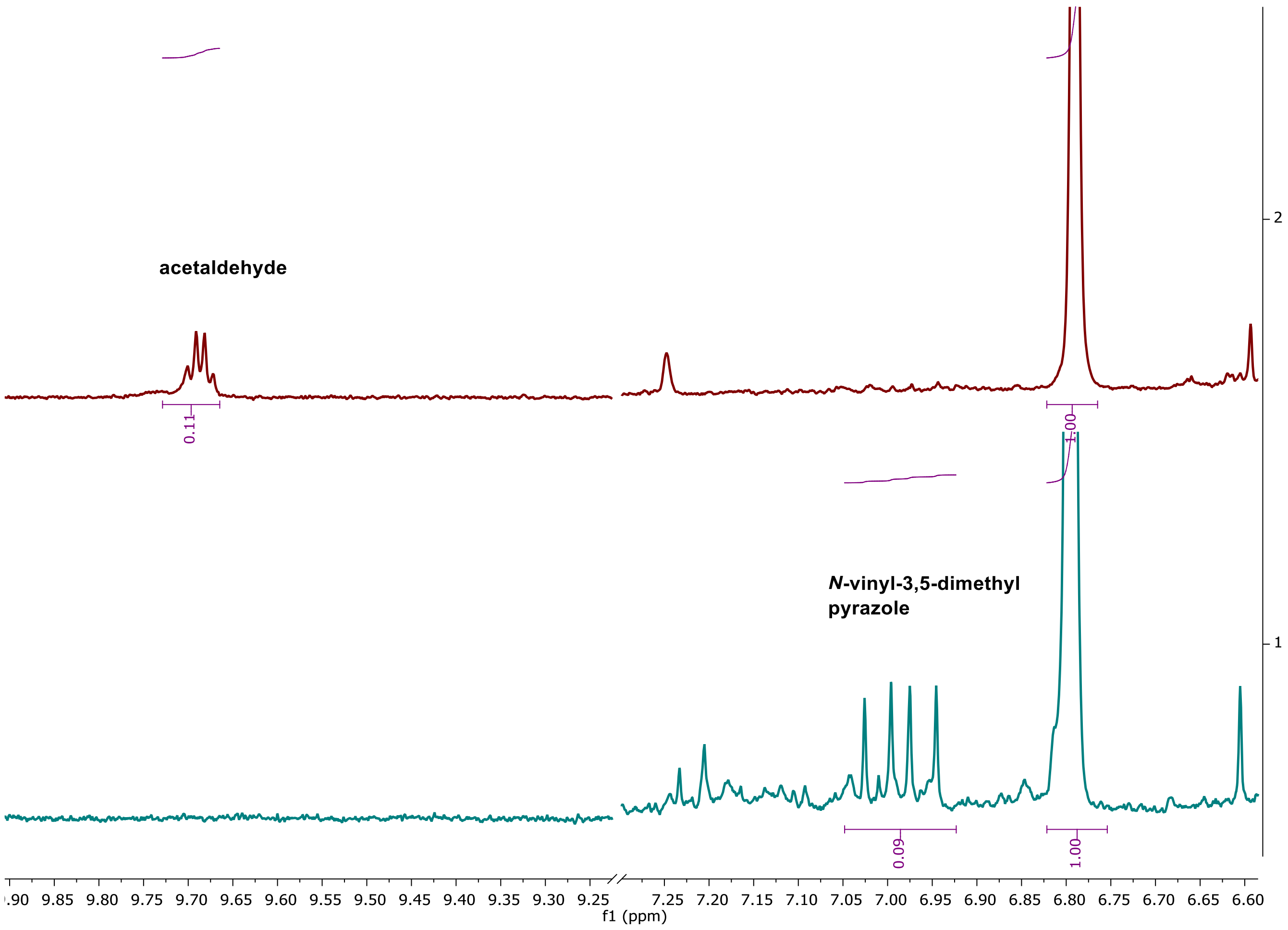

Fig. S18. Stacked ^{1}H NMR spectra of a reaction of $[WBr_2(pzH)_2(CO)_3]$ (**1**) and excess acetylene at room temperature for 24 h under exclusion of H_2O (bottom), and after the addition of excess water and heating to 60 °C for 24 h.

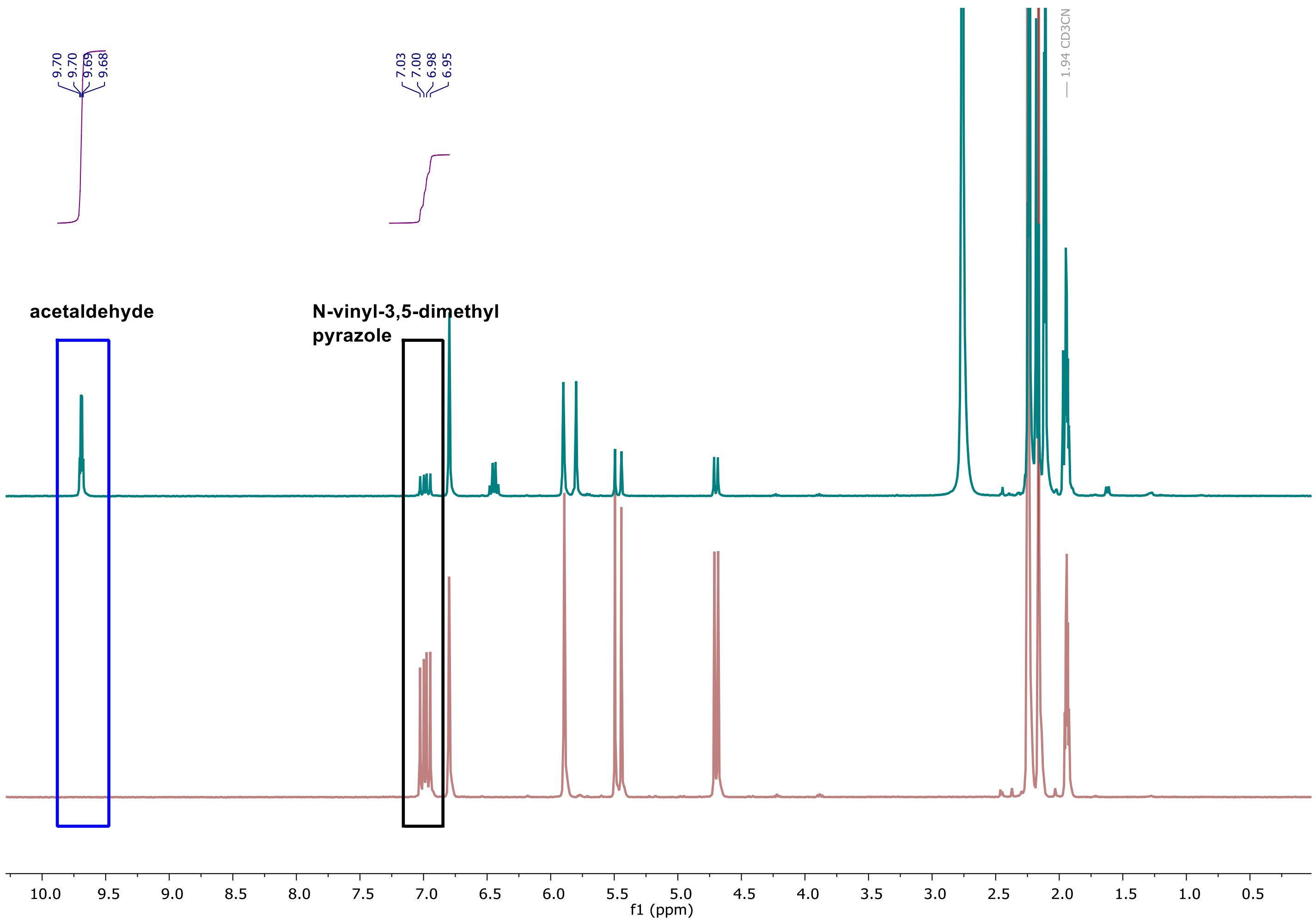

Fig. S19. Acid-catalyzed hydrolysis of *N*-vinyl-3,5-dimethyl pyrazole with aqueous HCl (10 % vs *N*-vinyl-3,5-dimethyl pyrazole) in CD_3CN at 60 °C after 0 h (bottom) and after 4 h (top).

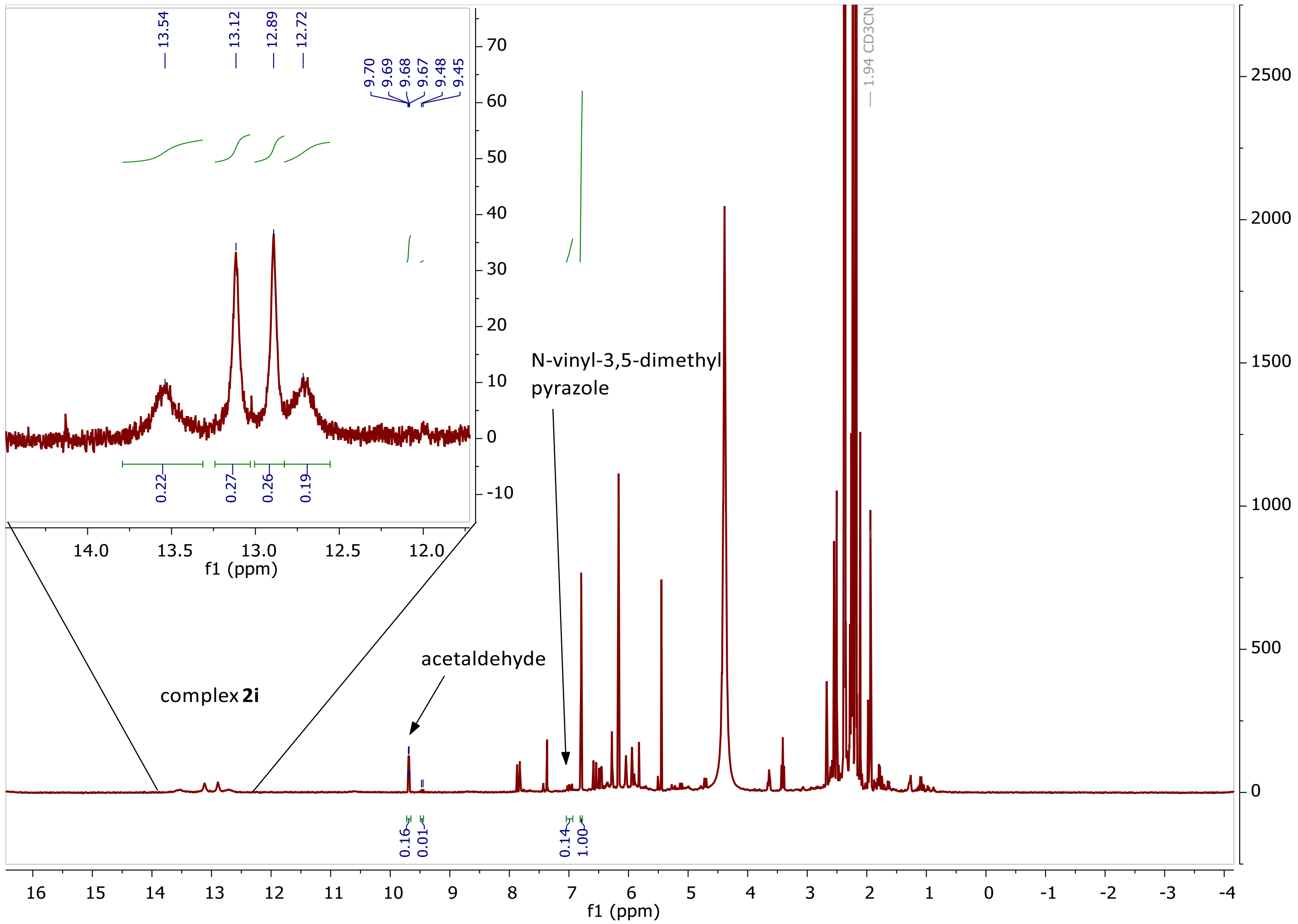

Fig. S20. ^{1}H NMR spectrum of a reaction of **1i** + 2 pzH + acetylene + H_2O (5 equiv) + mesitylene (1/3 equiv) in CD_3CN at 60 °C for 4 h. Shifts in the expanded view are consistent with those found in [WBr(C_2H_2)(pzH)(pz-NHCCH$_3$)(CO)]Br (**2i**) (Fig. S8).

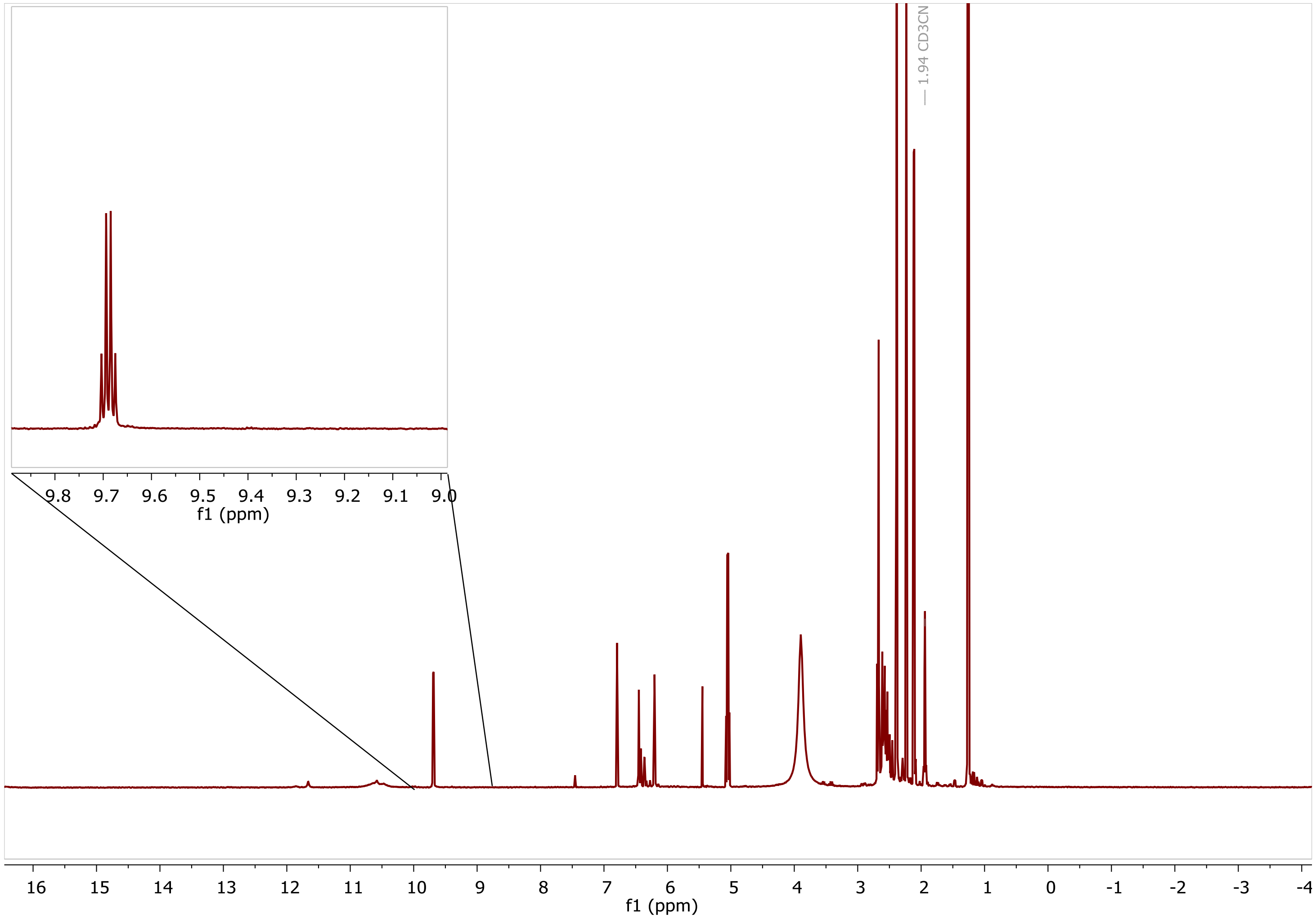

Fig. S21. ^{1}H NMR spectrum of a reaction of {**1i**+pzH} (prepared *in situ*) + H_2O (5 equiv) and acetaldehyde (2 equiv) heated to 60 °C for 4 h in CD_3CN. Crotonaldehyde signal at 9.46 ppm is absent.

5 Crystal structure determination

A single orange plate-shaped crystal of **1** and a single blue block-shaped crystal of **2i** were selected and mounted on a glass fiber on a Bruker APEX-II CCD diffractometer. A single orange plate-shaped crystal of **1i** and a dark violet block-shaped crystal of **3** were selected and mounted on a glass fiber on an XtaLAB Synergy, Dualflex, HyPix-Arc 100 diffractometer. All the measurements were performed using monochromatized Mo K_{α} radiation (0.71073 Å) at 100 K. Data reduction, scaling, and absorption corrections were performed for the four structures using CrysAlisPro (Rigaku, V1.171.43, 2023).[4] For structures **1** and **2i**, empirical absorption correction was performed using spherical harmonics, implemented in SCALE3 ABSPACK scaling algorithm. For **1i** and **3**, a numerical absorption correction based on gaussian integration over a multifaceted crystal model and an empirical absorption correction using spherical harmonics, implemented in SCALE3 ABSPACK scaling algorithm were performed.

The structures of **1**, **1i,** and **2i** were solved with the ShelXT 2018/2 structure solution program[5] using the intrinsic phasing solution method and by using Olex2[6] as the graphical interface. The models were refined with version 2019/3 of ShelXL[7] using full-matrix least-squares techniques against F^2. All non-hydrogen atoms were refined anisotropically. Hydrogen atom positions were calculated geometrically and refined using a riding model, except for the positions of the H atoms of the NH groups which were taken from a difference Fourier map, the N–H distances were fixed to 0.88 Å, and the H atoms were refined with individual isotropic displacement parameters without any constraints to the bond angles. The structure of **3** was solved with the ShelXT 2018/2 structure solution program[5] using the intrinsic phasing solution method and by using Olex2[6] as the graphical interface. The model was refined with version 2019/3 of ShelXL[8] using least squares minimization as a 2-component inversion twin with a BASF factor

of [0.505(14)]. All non-hydrogen atoms were refined anisotropically. Hydrogen atom positions were calculated geometrically and refined using a riding model except for the position of the H atom of the OH group, which was taken from a difference Fourier map and refined without constraint, and for the position of the H atom of the NH group which was taken from a difference Fourier map, the N–H distance fixed to 0.88 Å, and the H atom was refined with individual isotropic displacement parameters without any constraints to the bond angle. SADI, RIGU, and SIMU constraints and restraints were used to model the solvent in **3**. CCDC entries 2337531 (**1**), 2355110 (**1i**), 2337532 (**2i**), and 2337533 (**3**) contain the supplementary crystallographic data for this paper.

Table S2. Data collection and structure refinement details for compounds **1** and **1i**.

**Compound**	**1**	**1i**
CCDC Number	2337531	2355110
Empirical formula	$C_{13}H_{16}N_4O_3Br_2W$	$C_{10}H_{11}N_3O_3Br_2W \bullet CHCl_3$
Formula weight	619.97	684.26
Temperature /K	100.00(10)	99.97(18)
Crystal system	monoclinic	triclinic
Space group	$P2_1/n$	P-1
a /Å	10.5949(2)	9.2942(2)
b /Å	14.4051(3)	10.3885(2)
c /Å	11.7972(3)	11.1039(2)
α /°	90	63.402(2)
β /°	91.105(2)	75.4980(10)
γ /°	90	79.6940(10)
Volume /Å^3	1800.16(7)	925.34(4)
Z	4	2
ρ_{calc} g/cm^3	2.288	2.456
μ /mm^{-1}	10.874	11.007
F(000)	1160.0	636.0
Crystal size /mm^3	0.22 × 0.17 × 0.11	0.3 × 0.21 × 0.11
Radiation	Mo Kα (λ = 0.71073)	Mo Kα (λ = 0.71073)
2Θ range for data collection /°	4.464 to 56.56	4.54 to 61.012
Index ranges	-14 ≤ h ≤ 14, -19 ≤ k ≤ 19, -15 ≤ l ≤ 15	-13 ≤ h ≤ 13, -14 ≤ k ≤ 14, -15 ≤ l ≤ 15
Reflections collected	95310	28243
Independent reflections	4465 [R_{int} = 0.1121, R_{sigma} = 0.0281]	5632 [R_{int} = 0.0438, R_{sigma} = 0.0343]
Data/restraints/parameters	4465/2/220	5632/0/215
Goodness-of-fit on F^2	1.054	1.038
Final R indexes [I>=2σ (I)]	R_1 = 0.0343, wR_2 = 0.0824	R_1 = 0.0230, wR_2 = 0.0444
Final R indexes [all data]	R_1 = 0.0428, wR_2 = 0.0860	R_1 = 0.0267, wR_2 = 0.0452
Largest diff. peak/hole /e.Å^{-3}	3.70/-1.03	0.76/-0.96

Table S3.Data collection and structure refinement details for compounds **2i** and **3**.

**Compound**	**2i**	**3**
CCDC Number	2337532	2337533
Empirical formula	$C_{15}H_{21}Br_2N_5OW$	$C_{21}H_{29}BrN_6O_4W_2 \bullet CHCl_3$
Formula weight	631.04	996.48
Temperature /K	100.04(10)	100.00(13)
Crystal system	monoclinic	orthorhombic
Space group	$P2_1/c$	$P2_12_12$
a /Å	11.3229(3)	7.85700(10)
b /Å	8.0358(2)	15.9287(3)
c /Å	21.4613(5)	12.3215(2)
α /°	90	90
β /°	95.852(2)	90
γ /°	90	90
Volume /Å^3	1942.56(8)	1542.06(4)
Z	4	2
ρ_{calc} g/cm^3	2.158	2.146
μ /mm^{-1}	10.073	9.047
F(000)	1192.0	940.0
Crystal size /mm^3	0.2 × 0.15 × 0.1	0.163 × 0.075 × 0.051
Radiation	Mo Kα (λ = 0.71073)	Mo Kα (λ = 0.71073)
2Θ range for data collection /°	3.816 to 61.016	4.18 to 63.002
Index ranges	$-16 \le h \le 16, -11 \le k \le 11, -30 \le l \le 30$	$-11 \le h \le 11, -23 \le k \le 23, -18 \le l \le 17$
Reflections collected	159843	48614
Independent reflections	5931 [R_{int} = 0.0911, R_{sigma} = 0.0212]	5144 [R_{int} = 0.0383, R_{sigma} = 0.0221]
Data/restraints/parameters	5931/2/230	5144/43/203
Goodness-of-fit on F^2	1.044	1.062
Final R indexes [I>=2σ (I)]	R_1 = 0.0259, wR_2 = 0.0544	R_1 = 0.0220, wR_2 = 0.0457
Final R indexes [all data]	R_1 = 0.0342, wR_2 = 0.0571	R_1 = 0.0280, wR_2 = 0.0471
Largest diff. peak/hole /e.Å^{-3}	1.58/-0.68	1.33/-0.74

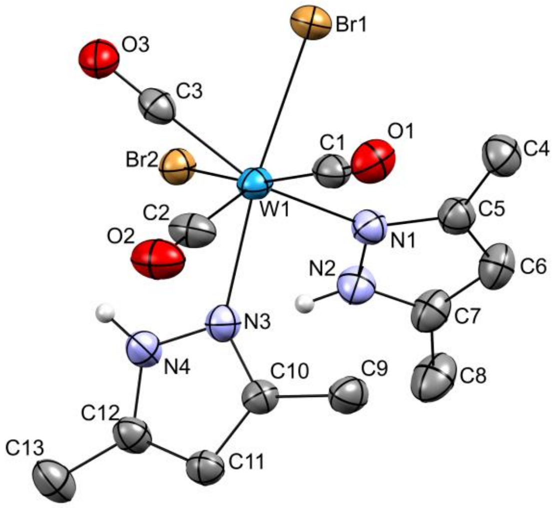

Fig. S22. Molecular structure of the asymmetric unit of $[WBr_2(pzH)_2(CO)_3]$ (**1**) showing the atomic numbering scheme. The probability ellipsoids are drawn at the 50% probability level. The H atoms were omitted for clarity except those of the pyrazole groups.

Table S4. Selected bond lengths (Å) and angles (°) in $[WBr_2(pzH)_2(CO)_3]$ (**1**)

W1-Br1	2.6347(5)	Br1-W1-Br2	86.849(17)
W1-Br2	2.6810(5)	C1-W1-Br1	79.31(15)
W1-C1	1.977(5)	C1-W1-Br2	164.96(16)
W1-C2	1.988(6)	C1-W1-C2	71.9(2)
W1-C3	1.976(6)	C1-W1-N1	89.57(18)
W1-N1	2.241(4)	C1-W1-N3	110.12(18)
W1-N3	2.230(4)	C2-W1-Br1	126.70(15)
C1-O1	1.153(6)	C2-W1-Br2	121.90(17)
C2-O2	1.160(7)	C2-W1-N1	134.7(2)
C3-O3	1.167(7)	C2-W1-N3	70.28(19)
		C3-W1-Br1	73.54(14)
		C3-W1-Br2	79.67(15)
		C3-W1-C1	101.8(2)
		C3-W1-C2	69.9(2)
		C3-W1-N1	155.46(19)
		C3-W1-N3	116.52(18)
		N1-W1-Br1	87.53(11)
		N1-W1-Br2	83.91(10)
		N3-W1-Br1	163.00(11)
		N3-W1-Br2	81.93(11)
		N3-W1-N1	78.65(16)
		O1-C1-W1	176.2(5)
		O2-C2-W1	177.9(6)
		O3-C3-W1	175.8(5)
		N2-N1-W1	119.0(3)
		C5-N1-W1	136.1(3)

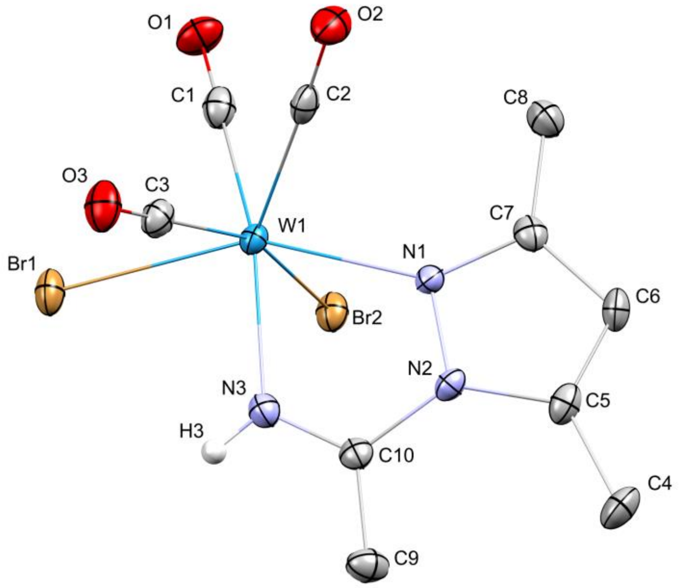

Fig. S23. structure of [WBr_2(pz-$NHCCH_3$)$(CO)_3$] (**1i**). The probability ellipsoids are drawn at the 50%. Except for the NH hydrogen, the H atoms and solvent molecules were omitted for clarity.

Table S5.Selected bond lengths (Å) and angles (°) in [WBr_2(pz-$NHCCH_3$)$(CO)_3$] (**1i**).

W1-Br1	2.6369(3)	Br1-W1-Br2	87.574(9)
W1-Br2	2.6707(3)	C1-W1-Br1	78.78(8)
W1-C1	2.010(3)	C1-W1-Br2	78.13(8)
W1-C2	1.973(3)	C1-W1-N1	119.12(10)
W1-C3	1.988(3)	C1-W1-N3	160.52(10)
W1-N1	2.185(2)	C2-W1-Br1	129.35(8)
W1-N3	2.164(2)	C2-W1-Br2	126.67(8)
C1-O1	1.148(3)	C2-W1-C1	74.64(11)
C2-O2	1.156(3)	C2-W1-C3	70.66(12)
C3-O3	1.141(3)	C2-W1-N1	77.43(10)
N2-C10	1.391(3)	C2-W1-N3	124.83(10)
N3-C10	1.273(4)	C3-W1-Br1	76.39(8)
C9-C10	1.487(4)	C3-W1-Br2	162.17(8)
		C3-W1-C1	105.78(12)
		C3-W1-N1	114.04(9)
		C3-W1-N3	82.62(11)
		N1-W1-Br1	152.66(6)
		N1-W1-Br2	77.26(6)
		N3-W1-Br1	86.43(7)
		N3-W1-Br2	88.74(6)
		N3-C1-N1	70.82(9)
		O1-C1-W1	176.9(3)
		O2-C2-W1	177.4(3)
		O3-C3-W1	179.6(2)
		N2-N1-W1	116.35(16)

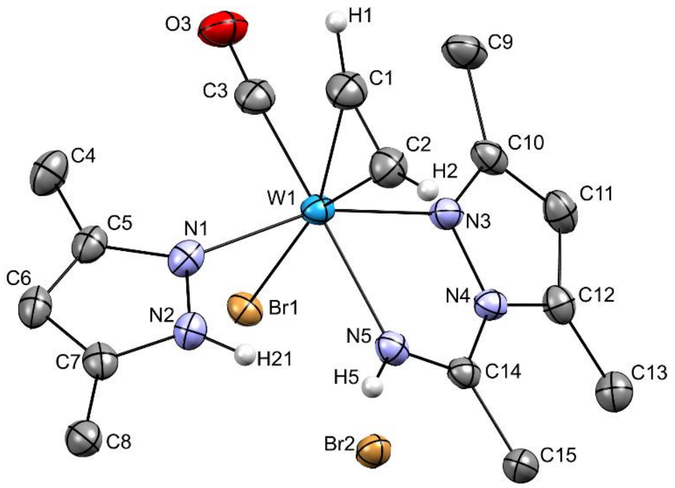

Fig. S24. Molecular structure of the asymmetric unit of [WBr(C_2H_2)(pzH)(pz-$NHCCH_3$)(CO)]Br (**2i**) showing the atomic numbering scheme. The probability ellipsoids are drawn at the 50% probability level. The H atoms were omitted for clarity except those of the ethyne group and those of the pyrazole groups.

Table S6. Selected bond lengths (Å) and angles (°) in [WBr(C_2H_2)(pzH)(pz-$NHCCH_3$)(CO)]Br (**2i**).

W1-C1	2.035(4)	C2-W1-C1	37.54(14)
W1-C2	2.009(3)	C2-C1-W1	70.1(2)
W1-Br1	2.6461(3)	C1-C2-W1	72.3(2)
W1-C3	1.972(4)	O3-C3-W1	176.3(3)
W1-N1	2.151(3)		
W1-N3	2.146(3)		
W1-N5	2.184(3)		
C1-C2	1.301(5)		
C3-O3	1.155(4)		
N5-C14	1.263(4)		
C14-C15	1.493(4)		

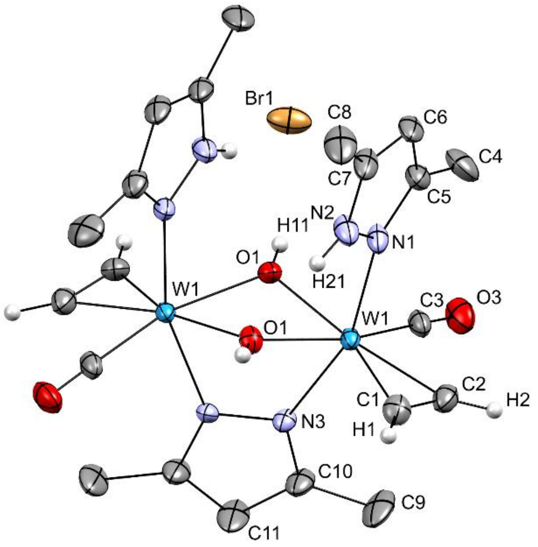

Fig. S25. Molecular structure of the asymmetric unit of [W(C_2H_2)(CO)(pzH)(μ-OH)$_2$(μ-pz)W(C_2H_2)(CO)(pzH)]Br (**3**) showing the atomic numbering scheme. The probability ellipsoids are drawn at the 50% probability level. The H atoms were omitted for clarity except those of the pyrazole groups, the hydroxyl groups, and the ethyne groups. The solvent molecule is omitted. The analysis shows that the complex lies on a C_2 rotation axis in the crystal. It is a cationic tungsten(II) dimer [W1-W1^1 3.343(1) Å] with a bromide counter anion. The two W centers are bridged by a μ-3,5-dimethylpyrazolyl ligand and two μ-hydroxido ligands with W-O bond lengths (W1-O1 2.056(5) Å, W1-O1^1 2.139(5) Å) close to the W-O bond found in AH, measured at 2.04 Å.[9] Each W center is surrounded by an η^2-acetylene ligand (C1-C2 1.297(8) Å, W1-C2 1.932(2) Å) and a carbonyl ligand *cis* and eclipsed to each other (C2-C1-W1-C3 2.453(4)°). A pyrazole ligand is coordinated per W center, with one of the NH groups forming a hydrogen bond with the bromide.

Table S7. Selected bond lengths (Å) and angles (°) in [W(C_2H_2)(CO)(pzH)(μ-OH)$_2$(μ-pz)W(C_2H_2)(CO)(pzH)]Br (**3**).

W1···W1^1	3.343(6)	O1-W1-O1^1	69.67(14)
W1-O1	2.056(5)	C1-W1-O1^1	88.3(2)
W1-O1^1	2.139(5)	C1-W1-O1	158.0(2)
W1-N1	2.163(3)	C1-W1-N1	95.1(3)
W1-N3	2.159(3)	C1-W1-N3	97.4(3)
W1-C1	2.025(7)	C1-W1-C2	37.1(2)
W1-C2	2.051(5)	C2-W1-O1^1	125.4(2)
W1-C3	1.956(6)	C2-W1-O1	164.8(2)
O3-C3	1.148(7)	C3-W1-O1	92.8(2)
C1-C2	1.297(8)	C3-W1-O1^1	162.1(2)
		C3-W1-C1	109.1(3)
		C3-W1-C2	72.0(3)
		W1-O1-W1^1	105.65(13)
		C2-C1-W1	72.5(4)
		C1-C2-W1	70.4(4)
		O3-C3-W1	178.4(8)

^{1} symmetry used for equivalent atoms: 1-x,1-y,+z

6 References

1 C. Vidovič, L. M. Peschel, M. Buchsteiner, F. Belaj and N. C. Mösch-Zanetti, *Chem. - Eur. J.*, 2019, **25**, 14267–14272.

2 P. K. Baker, M. E. Harman, S. Hughes, M. B. Hursthouse and K. M. Abdul Malik, *J. Organomet. Chem.*, 1995, **498**, 257–266.

3 R. Dator, A. Carrà, L. Maertens, V. Guidolin, P. W. Villalta and S. Balbo, *J. Am. Soc. Mass Spectrom.*, 2017, **28**, 608–618.

4 Rigaku, *CrysAlis Pro*, Rigaku, 2023.

5 G. M. Sheldrick, *Acta Crystallogr. A: Found. Adv.*, 2015, **71**, 3–8.

6 O. V. Dolomanov, L. J. Bourhis, R. J. Gildea, J. A. K. Howard and H. Puschmann, *J. Appl. Crystallogr.*, 2009, **42**, 339–341.

7 G. M. Sheldrick, *Acta Crystallogr. C Struct. Chem.*, 2015, **71**, 3–8.

8 C. B. Hübschle, G. M. Sheldrick and B. Dittrich, *J. Appl. Crystallogr.*, 2011, **44**, 1281–1284.

9 G. B. Seiffert, G. M. Ullmann, A. Messerschmidt, B. Schink, P. M. Kroneck and O. Einsle, *Proc. Natl. Acad. Sci. U.S.A.*, 2007, **104**, 3073–3077.
